# Determining the Different Mechanisms Used by *Pseudomonas* Species to Cope With Minimal Inhibitory Concentrations of Zinc via Comparative Transcriptomic Analyses

**DOI:** 10.3389/fmicb.2020.573857

**Published:** 2020-12-03

**Authors:** Lei Lei, Jiahui Chen, Weifang Liao, Pulin Liu

**Affiliations:** College of Biological and Pharmaceutical Engineering, Wuhan Polytechnic University, Wuhan, China

**Keywords:** *Pseudomonas*, zinc, comparative analysis, transcriptomic, minimal inhibit concentration

## Abstract

*Pseudomonas* is one of the most diverse bacterial genera identified in the environment. Genome sequence analysis has indicated that this genus can be clustered into three lineages and ten groups. Each group can adopt different mechanisms to thrive under zinc-depleted or high-zinc conditions, two environments that are frequently encountered during their environmental propagation. The response of three prominent *Pseudomonas* strains (*Pseudomonas aeruginosa* PAO1, *Pseudomonas putida* KT2440, and *Pseudomonas fluorescens* ATCC 13525^T^) to minimal inhibitory concentrations of zinc were compared using RNA-seq and ultra-performance liquid chromatography–tandem mass spectrometry analysis. Results demonstrated that the three strains shared only minimal similarity at the transcriptional level. Only four genes responsible for zinc efflux were commonly upregulated. *P. aeruginosa* PAO1 specifically downregulated the operons involved in siderophore synthesis and the genes that encode ribosomal protein, while upregulated the genes associated with antibiotic efflux and cell envelope biosynthesis. The membrane transporters in *P. putida* KT2440 were globally downregulated, indicating changes in cell permeability. Compared with *P. aeruginosa* PAO1 and *P. putida* KT2440, the most remarkable transcriptional variation in *P. fluorescens* ATCC 13525^T^ is the significant downregulation of the type VI secretion system. Metabolite quantitative analysis showed that low concentrations of the metabolites involved in central carbon metabolism and amino acid synthesis were detected in the three strains. In summary, the cellular responses of the three strains under high-zinc condition is quite divergent. Although similar metal efflux systems were upregulated, the three strains employed different pathways to reduce zinc intrusion. In addition, zinc treatment can increase the difficulties of scavenging *P. aeruginosa* from its colonization area, and reduce the competitiveness of *P. fluorescens* in microbiota.

## Introduction

Zn^2+^ is the second most abundant essential metal ion after Fe^2+/3+^ in all three kingdoms of life, which directly interacts with RNA polymerase, zinc-finger proteins and superoxide dismutase to maintain their functionality (Hobman et al., 2007; Watły et al., 2016; Gonzalez et al., 2018). Free Zn^2+^ in the cytoplasm is normally maintained at the femtomolar to picomolar level (Outten and O'Halloran, 2001). Once this threshold is surpassed, excess Zn^2+^ can replace other metal ions from proteins and alter the stability of biomolecules (Krezel and Maret, 2016; Peng et al., 2018). Although zinc is a redox-inactive metal under normal conditions, high levels of intracellular zinc clearly induce the generation of reactive oxygen species (ROS) as evidenced by the increased amount of oxidized protein (Alhasawi et al., 2014). In nature, the geological background level of zinc is low (Cheng, 2003). However, anthropogenic industries and domestic activities directly or indirectly release vast amounts of Zn^2+^ into the environment. In developing countries, mining activity coupled with poor effluent disposal and management often caused severe zinc pollution in soils or sediments. For instance, Vasile et al. (2008) analyzed the zinc partition in sediments taken from a river subjected of intense mining activities, and found that the easily soluble zinc ranged from 80 to 300 mg Kg^−1^. Klimek (2012) reported that the averaged concentration of water soluble zinc in the forest soils surrounded by industrial area achieved 82.30 mg Kg^−1^. With the progress of urbanization, large numbers of municipal wastewater treatment plants have been built. The sewage sludge is another material that accumulates zinc ions. Tu et al. (2012) used a four-step extraction method to extract heavy metals in municipal sludges from seven individual wastewater treatment plants. The results showed that zinc in some sludges is highly bioavailable (the water soluble zinc concentration reached 61.42±7.0 mg Kg^−1^). Fertilizing use of these sludges may cause severe zinc pollution in soils.

Prokaryotes are single cell organisms that have direct contact with their surrounding environment. Thus, these organisms are extremely vulnerable to environmental stresses (Blencowe and Morby, 2003). In response to metal stress, many bacteria have evolved numerous mechanisms, including mobilization, precipitation, biosorption and biogeochemical cycling, to respond to the adverse effects of metal ions (Singh et al., 2018). *Pseudomonas* is a ubiquitous bacterial genus with high adaptability to various environments (Santos et al., 2004). Many *Pseudomonas* species exhibit high capability to maintain the equilibrium of cytoplasmic metal ions, which directly affects metal availability and distribution (Haritha et al., 2008; Ramos and Filloux, 2010). In our previous study, the transcriptional responses of *P. putida* KT2440 to low, intermediate, and high levels of extracellular zinc stresses were thoroughly analyzed (Peng et al., 2018). The results showed that genes associated with metal transport and membrane homeostasis were significantly induced at the lowest zinc level. As zinc concentrations increased, the respiration chains, amino acids and carbon source metabolisms, as well as the biogenesis of Fe-S clusters were adjusted. However, *Pseudomonas* comprises ~200 species (Kohlstedt and Wittmann, 2019), which exhibit considerable diversity in their ecological habitat and physiology (Peix et al., 2017; Singh et al., 2018). Recent phylogenetic analysis has clustered *Pseudomonas* into three lineages and ten groups (Peix et al., 2017). Each *Pseudomonas* group probably uses different way to alleviate metal-induced intracellular disorder. A comparison of our transcriptome data with the results obtained by Alhasawi et al. (2014) showed partial differences between the response of *P. putida* and *P. fluorescens* to zinc. For example, the expression of NADPH-generating enzymes, including isocitrate dehydrogenase-NADP^+^, malic enzyme (ME), and glucose-6-phosphate dehydrogenase (G6PDH), was markedly increased in *P. fluorescens*. Meanwhile, the most upregulated NADPH-generating enzyme in *P. putida* KT2440 was ferredoxin-NADP^+^ reductase and the transcription of ME and G6PDH remained unchanged. In addition to *P. fluorescens* and *P. putida, P. aeruginosa* represents another important phylogenetic group in the *Pseudomonas* genus (Jensen et al., 2004; Peix et al., 2017). As a widespread environmental bacterium and an opportunistic animal pathogen, *P. aeruginosa* also encounters changing levels of zinc concentrations (Pederick et al., 2015). To date, studies on zinc homeostasis in *P. aeruginosa* mainly concentrated on zinc efflux and zinc associated virulence or antibiotic resistance (Braymer and Giedroc, 2014; Pederick et al., 2015; Gonzalez et al., 2018), other cellular responses remain poorly understood.

In the current study, three prominent *Pseudomonas* strains (*P. putida* KT2440, *P. aeruginosa* PAO1, and *P. fluorescens* ATCC 13525^T^) were challenged with external zinc at minimal inhibitory concentrations (MICs). Their responses were compared to provide a comprehensive understanding of the genes, the cellular processes and the cellular molecules that play crucial roles in zinc adaptation.

## Materials and Methods

### Bacterial Strains, Growth Conditions, and MIC Value Determination

*P. putida* KT2440 (ATCC 47054), *P. aeruginosa* PAO1 (ATCC 47085), and *P. fluorescens* ATCC 13525^T^ were obtained from American Type Culture Collection (ATCC, Manassa, Virginia, USA). These strains were routinely grown in a semi-synthetic cation-defined medium (CDM) (Pederick et al., 2015) at their optimum growth temperatures (30°C for *P. putida* KT2440 and *P. fluorescens* ATCC 13525^T^, 37 °C for *P. aeruginosa* PAO1). The MIC values of zinc ions were determined as described by Mergeay et al. (1985). The exponential cells of the three strains were serially diluted and then spread onto CDM agar plates containing zinc sulfate from 0.2 mmol L^−1^ to 2.0 mmol L^−1^ (0.1 mmol L^−1^ interval). The MIC values were defined as the lowest concentrations of zinc sulfate at which no colonies were observed after 48 h of incubation at optimum temperature. For zinc treatment, the exponential cells grown in the CDM culture were harvested via centrifugation and inoculated into new CDM containing zinc sulfate at MIC to achieve a cell density of 10^7^ CFU mL^−1^. After zinc treatment for 1 h, the cells were stabilized using RNA protect bacterial reagent (Qiagen, Valencia, CA, USA) at a ratio of 2:1 (2 mL of reagent per 1 mL of culture). The control groups were grown in CDM without zinc. Three biological replicates were performed for each treatment.

### RNA Extraction and RNA-seq Analysis

The total RNAs was extracted separately from each replicate using Trizol reagent (Invitrogen, Carlsbad, CA, USA). Residual genomic DNA contamination was removed from the total RNA samples via DNAase I digestion. The quality and quantity of total RNA was measured using an Agilent 2100 Bioanalyser. RNA-seq libraries were prepared and sequenced at the Beijing Genomics Institute (Beijing, China). 150 bp pair-end reads were sequenced using an Illumina HiSeq 2500 platform (Illumina, San Diego, CA, USA).

Raw data generated from Illumina sequencing platform were trimmed using Skewer version 0.2.2 (Jiang et al., 2014). After Quality control (Peng et al., 2018), Bowtie 2 version 2.2.3 (Langmead and Salzberg, 2012) was used to align the remaining reads to the *Pseudomonas* genomes (Winsor et al., 2015). The RPKM (reads per kilobase of transcript per million mapped reads) values were used for calculating gene expression level using DESeq2 version 2.2.2 (Love et al., 2014). DESeq generated all pairwise comparisons of treatments and associated adjusted *P*-values (P_adj_) controlling for the false discovery rate. Genes with average absolute fold changes higher than 2.0 and P_adj_ values <0.01 were classified as differentially expressed genes (DEGs). The functional clusters of orthologous groups classification of all DEGs was performed automatically using the Integrated Microbial Genomes portal (Markowitz et al., 2013).

### Validation of RNA-seq Results via RT-qPCR

To verify the validity of the RNA-seq data, RT-qPCR was performed with the same RNA samples used for RNA-seq analysis. Six genes were selected from each *Pseudomonas* strain. Therefore, a total of 18 genes were used for validation. The gene-specific primer pairs were designed using Beacon designer (Supplementary Table 1). The expression levels of the selected genes were calculated through the 2^−ΔΔ*Ct*^ method (Livak and Schmittgen, 2001). For cDNA analysis, 1 μg of RNA was reverse-transcribed using PrimeScript RT reagent kit (Takara, Dalian, China) and random hexamer primers following the manufacture's protocol. The quantities of cDNA were analyzed using the qPCR-SYBR Premix ExTaq (Takara, Dalian, China) in a Bio-Rad iCycler (Bio-Rad, Berkeley, CA, USA). PCR conditions were 95°C for 1 min, followed by 40 cycles of 95°C for 15 s, annealing temperature for 15 s, and 72°C for 15 s. A melting curve analysis was then performed at 95°C for 15 s, 60°C for 30 s, and 95°C for 15 s. Shapiro-Wilk test (SPSS 20.0, Chicago, IL, USA) was conducted for the fold-change values obtained from the RNA-seq and RT-qPCR (Gómez-Sagasti et al., 2015). Given that all data followed a normal distribution, Pearson's coefficients were calculated to illustrate the correlation level between two data groups.

### Metabolite Extraction and UPLC-MS/MS Analysis

The bacterial samples used for the metabolome analysis were prepared as described by Chavez-Dozal et al. (2015). Zinc-treated *Pseudomonas* cells were collected from each biological replicate via centrifugation at 10,000 × g for 5 min at 4°C. After washing two times with ice-cold phosphate-buffered saline (pH 7.4), the cell pellets were frozen with liquid nitrogen. To extract the metabolites, 50 mg of the frozen samples were dispersed in 1 mL of liquid containing methanol/acetonitrile/water (2:2:1), sonicated for 10 min at 0°C, and then centrifuged at 14,000 × g for 20 min at 4°C. The resulting supernatants were lyophilised and reconstituted in 100 μL of acetonitrile/water (1:1) before UPLC-MS/MS analysis.

An Agilent 1290 HPLC system (Agilent, Santa Clara, CA, USA) equipped with an HILIC analytical column (amide 1.7 μm, 2.1 × 100 mm, Waters, Milford, MA, USA) was used to analyse the extract of each sample. The mobil phase was water (containing 25 mmol L^−1^ CH_3_COONH_4_ and 25 mmol L^−1^ ${\text{NH}}_{3}^{.}$H_2_O, Solvent A), and acetonitrile (Solvent B). The dilution programme was operated for 0.5 min 95:5 (B:A) then switched to 40:60 for 8.5 min before returning to 95:5 for 9 min further. The mass spectra were recorded by an Agilent 6540 Q-TOF mass spectrometer operating in the positive and negative ion modes using an Agilent electrospray ionization source. After each spectrum was normalized to the total spectral intensity, the profile data were imported into SIMCA-P^+^ software (Umetrics AB, Umeå, Sweden) and pareto-scaled to perform orthogonal partial least squares-discriminate analysis (OPLS-DA). The significance of the metabolites was ranked using the variable importance in projection score from the OPLS-DA model. For the univariate analysis of a specific metabolite, statistical significance was determined using Student's two-sample *t*-test. The metabolites with average absolute fold changes higher than 2.0 and P_adj_ values <0.05 were defined as statistically different.

## Results

### MICs of Zinc Sulfate

Given that metal ions exert does-dependent effects on bacterial cells, comparing the cellular responses of different strains at the same zinc stress level is important. In this study, the MIC of ZnSO_4_ was used in the treatment. *P. putida* KT2440 was highly tolerant to zinc and exhibited a zinc MIC value of up to 1.1 mmol L^−1^. *P. fluorescens* was the least zinc-tolerant strain with an MIC value of approximately 0.4 mmol L^−1^. *P. aeruginosa* PAO1 had intermediate levels of zinc tolerance. A cation-defined medium (CDM) plate that contained more than 0.7 mmol L^−1^ zinc completely inhibited its colony formation. Therefore, in the following RNA-seq and UPLC-MS/MS analysis, 0.7, 1.1, and 0.4 mmol L^−1^ zinc sulfate were used to compare the zinc-induced cellular responses in *P. aeruginosa* PAO1, *P. putida* KT2440, and *P. fluorescens* ATCC 13525^T^.

### Global Transcription Features of the Three *Pseudomonas* Strains

**Figure 1:**
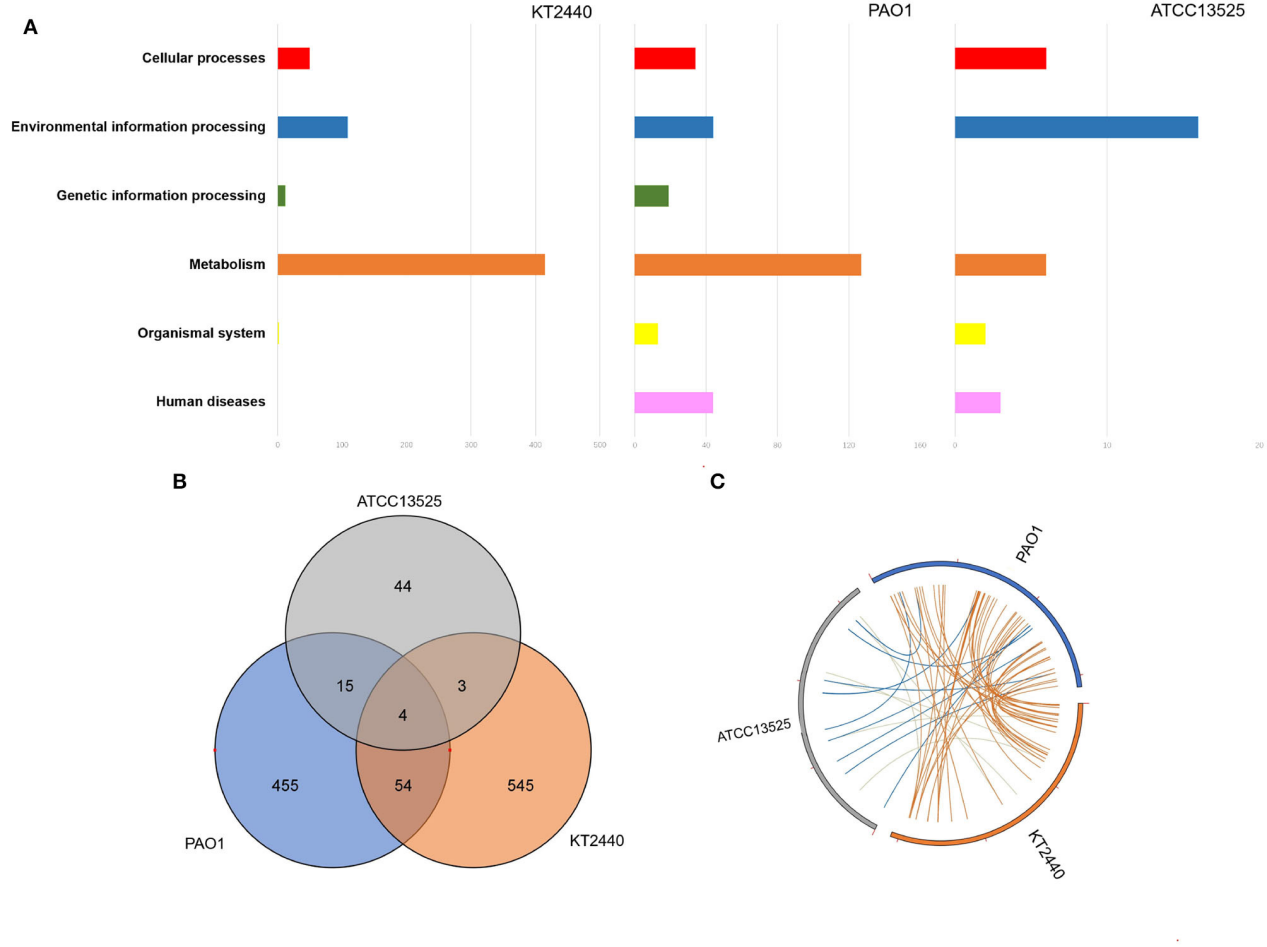
Global transcription features of three *Pseudomonas* strains. **(A)** KEGG analysis of DEGs identified in *P. aeruginosa* PAO1, *P. putida* KT2440, and *P. fluorescence* ATCC 13525^T^. **(B)** Venn diagram showing the DEGs commonly or strain-specifically regulated by these three *Pseudomonas* strains. **(C)** Genome location of these genes regulated by more than one strain.

The numbers of differentially expressed genes (DEGs) during the zinc treatment of *P. aeruginosa* PAO1, *P. putida* KT2440, and *P. fluorescens* ATCC 13525^T^ are provided in Table 1. The comparison of the zinc-treated *P. aeruginosa* PAO1 with their control samples identified 529 genes that were differently transcribed. Amongst which, 406 genes, 7.1% of the total coding DNA sequence (CDS) in the genome, were upregulated, whereas, 123 (2.2%) genes were downregulated. The comparison of the zinc-treated *P. putida* KT2440 with their control bioreplicates showed that, the expression of 607 genes was significantly altered, with the majority (344 genes, 6.0%) displaying significant induction in transcription compared with those (263 genes, 4.6%) demonstrating decreased transcription. The lowest number of DEGs was observed in *P. fluorescens* ATCC 13525^T^, with only 48 induced genes and 19 repressed genes (Table 1). In general, KEGG analysis showed that large quantities of DEGs identified in *P. aeruginosa* PAO1 and *P. putida* KT2440 can be clustered into the metabolism class (Figure 1A), particularly the carbohydrate and energy metabolism subclasses. As an opportunistic animal pathogen, *P. aeruginosa* PAO1 also regulated many genes belonging to the human disease class. The cellular response of *P. fluorescens* ATCC 13525^T^ differed from those of *P. aeruginosa* and *P. putida*, in which ~50% of DEGs with clear KEGG classifications were categorized into the environmental information processing group. To better compared the cellular responses occurred in the three *Pseudomonas* strains, the DNA sequence of the DEGs was extracted from each genome, and then blast against the DEGs identified in the other two genomes with a match cutoff of 70% and an E-value exponent cutoff of 1-e5. As shown in Figure 1B, only four orthologs were commonly regulated by the three *Pseudomonas* strains, and 1044 genes were strain-specifically regulated. The transcriptional patterns of *P. aeruginosa* and *P. putida* presented high similarities with 58 commonly regulated DEGs. In accordance with the KEGG analysis, *P. fluorescens* stands out in in all pair-wise blast analysis. Only 19 orthologs were commonly regulated by *P. fluorescens* and *P. aeruginosa*, and this value was reduced to 7 in *P. fluorescens* vs. *P. putida* analysis.

**Table 1:** The quantity of DEGs in *P. aeruginosa* PAO1, *P. putida* KT2440, and *P. fluorescens* ATCC 13525^T^.

**Strain**	**Upregulated**	**Downregulated**	**Combined (%)^**a**^**
*P. aeruginosa* PAO1	406	123	9.3
*P. putida* KT2440	344	263	10.6
*P. fluorescens* ATCC 13525^T^	48	19	1.14

a *Percentage of the protein encoding genes with changes more than 2-fold*.

### Functional Analysis of DEGs Identified in More Than One Strain

All the four DEGs (*cadA, cadR, czcR*, and *czcS*) commonly upregulated by the three strains were involved in metal efflux. *cadA* has been recognized as an efficient P-type ATPase that moves several divalent heavy metal ions out of the cellular membrane (Leedjärv et al., 2008), whilst *czcRS* has been proven as an important two-component system that connects metal efflux and porins. In *P. aeruginosa*, unphosphorylated CzcR decreased the expression of outer membrane porins, OprD. By contrast, the presence of phosphorylated CzcR induced the most effective metal efflux system, *czcCBA* (Perron et al., 2004). DEGs mutually regulated by *P. aeruginosa* PAO1 and *P. putida* KT2440 are listed in Supplementary Table 2. As expected, *czcCBA* operons, which are known to be induced by several divalent metal ions, were found to be highly responsive. The analysis of other DEGs with known functions showed that most of these DEGs can be manually clustered into three categories. The genes involved in membrane structure and channels constituted the largest functional category. In this group, seven genes, including *desA, ompB, dgkA, warB, warA, amgR* and PA4819 (PP_0034), were strongly upregulated (>5 fold), whereas *oprD* and *kguT* were mutually downregulated. In the other two groups, genes associated with central metabolism and those responsible for protein folding and degradation were generally upregulated. For *P. fluorescens*, the most prominent response mutually identified in *P. fluorescens* and *P. aeruginosa* was the upregulation of acyl-CoA dehydrogenase (RS07420, RS06160) and protein-tyrosine-phosphatase (RS10450). A ton-dependent receptor (RS23050) and a non-ribosomal peptide synthatase were markedly down-regulated (RS27315) (Supplementary Table 3). In addition to the four DEGs commonly regulated by the three strains, *P. fluorescens* and *P. putida* shared three more transcripts (Supplementary Table 4). Two of them were responsible for active transmembrane transport (RS21740, RS19005). The induction of asparaginase (RS29965) was also identified. The genome locations of these genes that are regulated by more than one strain are spread throughout the genomes, no obvious genomic island was observed (Figure 1C).

### DEGs Specifically Identified in *P. aeruginosa* PAO1

The DEGs specifically identified in *P. aeruginosa* PAO1 are shown in Supplementary Table 5. The fold-change data indicated that more energy were required for *P. aeruginosa* to alleviate zinc-induced toxicity. The proton-translocating NADH:ubiquinone oxidoreductase complex (*nuoGHIJLMN*) and the pyruvate dehydrogenase complex (PA3416-3417), which transforms pyruvate into acetyl-CoA were upregulated by ~2.3-fold, whilst the synthesis of ribosomal protein was significantly downregulated (*rpsB, rpsS, rpsT, rpsR, rpsF, rplS, rplW, rplJ*, and *rplU*). A distinct group of operons involved in lipid A synthesis/modification was highly induced (*arnBCADEF*), suggesting that the *P. aeruginosa* cells were in a cell-envelope-stressed state. In support of this, there was also significant upregulation of other genes previously linked to bacterial cell envelope homeostasis, such as *glmM* (phosphoglucosamine mutase) and *pagL* (lipid A 3-O-deacylase). The direct replacement of iron from their binding sites has been recognized as an important mechanism for zinc to exhibit cytotoxicity. Therefore, it is reasonable that the transcription of *pvdQAPNOFEDJ* that responsible for pyoverdine biosynthesis was downregulated. Using UV-visible spectrophotometry analysis (Hoegy et al., 2014), the concentration of pyoverdine presenced in the cultures was also determined. The biosynthesis of pyoverdine in zinc treated *P. aeruginosa* PAO1 (40.1 μmol L^−1^) reduced about 66.8% compared with control samples (121.4 μmol L^−1^) (Supplementary Figure 1).

Given that *P. aeruginosa* is an opportunistic pathogen, monitoring the transcriptional variation of pathogenicity related genes is important. The MIC of zinc strongly enhanced the transcription of *mexRAB-oprM* (Poole et al., 1996) and *mexXY* (Morita et al., 2012), two important multidrug efflux systems responsible for antibiotic resistance. Moreover, one operon involved in alginate synthesis (*algU-mucABC*) was significantly activated. The overproduction of exopolysaccharide alginate caused mucoid conversion in *P. aeruginosa* (Mathee et al., 1999; Rao et al., 2011), which increased bacterial metal tolerance via metal chelation (Gómez-Sagasti et al., 2015). Amongst all the DEGs specifically identified in *P. aeruginosa*, the most upregulated gene was *ptrA*. The function of PtrA was studied by two groups, but contrasting conclusions were reported. One group showed that PtrA directly binds to ExsA, which in turn, suppresses the expression of the type III secretion system (T3SS) (Ha et al., 2004). By contrast, the other group demonstrated that PtrA is a periplasmic protein, the expression of which increases the Cu tolerance of *P. aeruginosa* without affecting basal ExsA (Elsen et al., 2011). Our data supported that PtrA is not a T3SS repressor because the transcription of the T3SS gene clusters remained unchanged when *ptrA* was considerably induced by zinc.

### DEGs Specifically Identified in *P. putida* KT2440

In our previous study, the transcriptional response of *P. putida* KT2440 to stress-inducing concentrations of zinc was analyzed. The results showed that different zinc stress levels strongly influenced (>4 fold change) the transcription of genes from four functional groups, including metal transporting genes, genes associated with membrane homeostasis, antioxidant-encoding genes and genes involved in basic cellular metabolism (Peng et al., 2018). In this study, a twofold change was used as a criterion, and a total of 545 genes were identified as DEGs specifically regulated in *P. putida* KT2440. Approximately 64% of the downregulated operons encode nutrient uptake transporters (Supplementary Table 6), which indicated that *P. putida* KT2440 decreased the permeability of the cell envelope under zinc treatment. Only the transporters responsible for methoine, sulfate, and sulfonates uptake were upregulated, suggesting that, unlike *P. aeruginosa* PAO1, *P. putida* KT2440 did not suffer from a severe iron metabolism perturbation, but the sulfur-containing molecules were considerably disrupted. The synthesis of ribosomal protein was another remarkable difference between *P. aeruginosa* PAO1 and *P. putida* KT2440. In *P. aeruginosa* PAO1, the transcription of ribosomal protein encoding genes was generally downregulated. Meanwhile, their orthologs in *P. putida* KT2440 were highly stable, and even the transcription of *rpmH* was increased. In addition, the MIC of zinc significantly increased the transcription of the nickel (*nikABCDE*) and arsenic (*arsBR*) efflux systems in *P. putida* KT2440. Such phenomenon was not observed in the other two strains.

### DEGs Specifically Identified in *P. fluorescens* ATCC 13525^T^

Compared with the transcriptional responses of *P. aeruginosa* PAO1 and *P. putida* KT2440, the downregulation of type VI secretion system (T6SS) was the most remarkable feature of *P. fluorescens* ATCC 13525^T^ under zinc stresses (Supplementary Table 7). T6SS is widely distributed across diverse bacterial species; around one-third of the sequenced Gram-negative bacteria possess T6SS-associated genes (Alteri and Mobley, 2016). Bacterial T6SS functions as a contractile nanomachine that delivers effectors upon direct contact with a target cell (Drebes Dörr and Blokesch, 2018). Given that these effectors have different functions but frequently disturb the cellular structure, such as the cell wall, nucleic acid, or membrane compartment, T6SS is mainly perceived as an antibacterial weapon (Alteri and Mobley, 2016). In recent studies, the T6SSs in *Burkholderia thailandensis* and *Yersinia pseudotuberculosis* were found to secrete metal binding proteins which facilitate the intracellular accumulation of Zn^2+^ (Wang et al., 2015; Si et al., 2017). Similar capability of ion transport by T6SS was also found in *P. aeruginosa*. Its T6SS effector, TseF, facilities the delivery of outer membrane vesicles associated iron to bacterial cells by engaging the Fe-pyochelin receptor FptA and the porin OprF (Lin et al., 2017). Based on these published results and the RNA-seq results obtained in this study, we infer that T6SS also plays an important role in zinc transport in *P. fluorescens*, the downregulation of which reduced the intrusion of zinc across the outer membrane.

### Reverse Transcription Quantitative Real-Time Polymerase Chain Reaction (RT-qPCR) Validation

**Figure 2:**
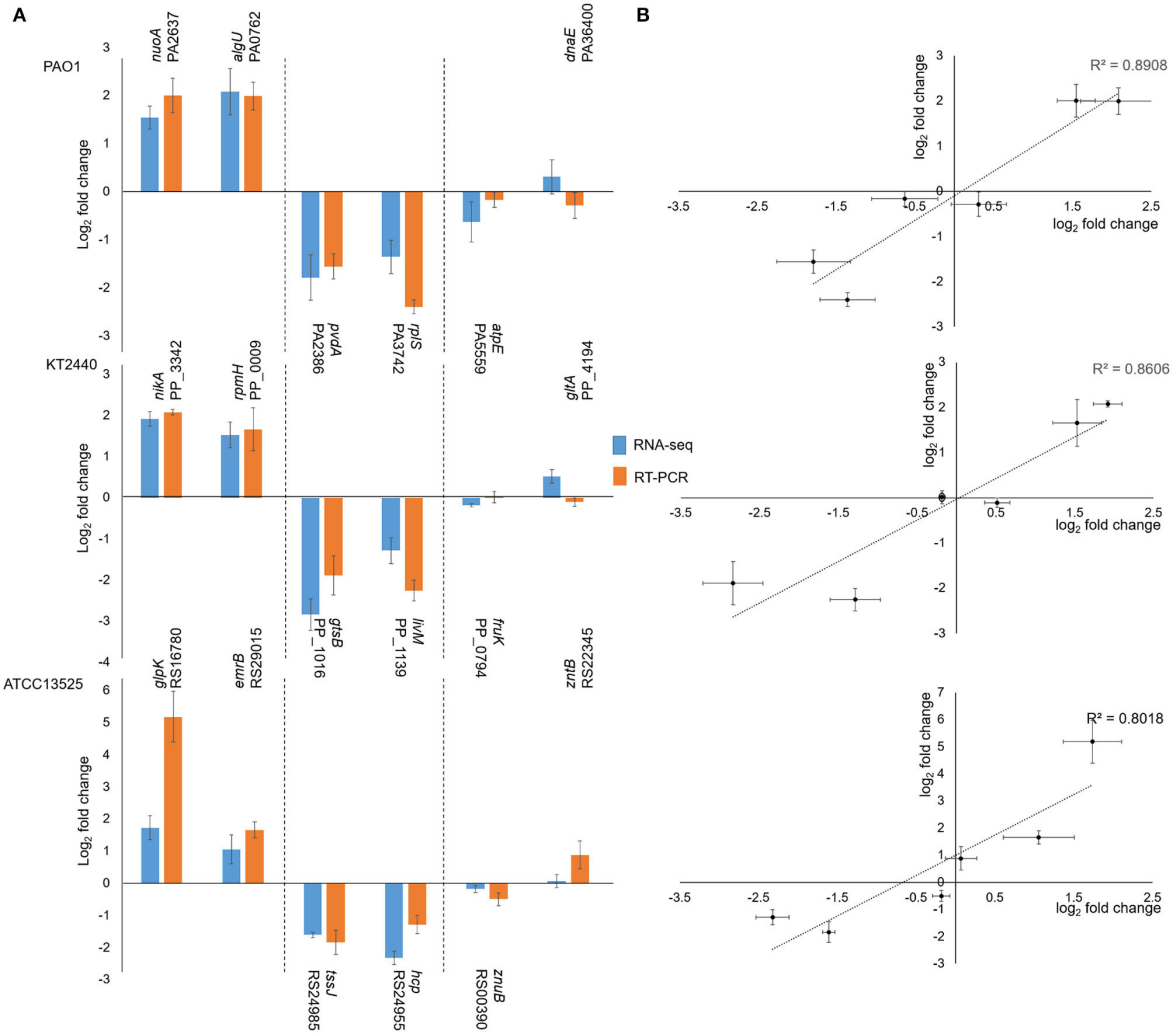
Correlation between the results obtained by RNA-seq and RT-PCR. **(A)** Rectangles represent fold changes of selected genes. Bars illustrate standard deviations of three replicates. **(B)** Correlation plot between fold change values of selected genes observed by RNA-seq and RT-PCR. Horizontal bars represent standard deviations for RNA-seq data, and vertical bars illustrated standard deviations of RT-PCR results. As the fold change values followed the normal distribution, Pearson' coefficients were calculated.

To confirm the RNA-seq data, six genes were selected from each strain, and their transcription was analyzed via RT-qPCR with three biological replicates. As shown in Figure 2, the dynamic transcription patterns of all the genes were consistent with the RNA-seq data (Figure 2A), and the observed fold changes for each gene were moderately correlated (*R*^2^ ranged from 80.1 to 89.1%) (Figure 2B). Therefore, the RT-qPCR results validated the accuracy of the RNA-seq data. Moreover, previous studies on the transcriptomic profiles of *P. putida* KT2440 were useful in further confirming the results obtained in this study.

### Metabolite Changes in the Three *Pseudomonas* Strain

**Figure 3:**
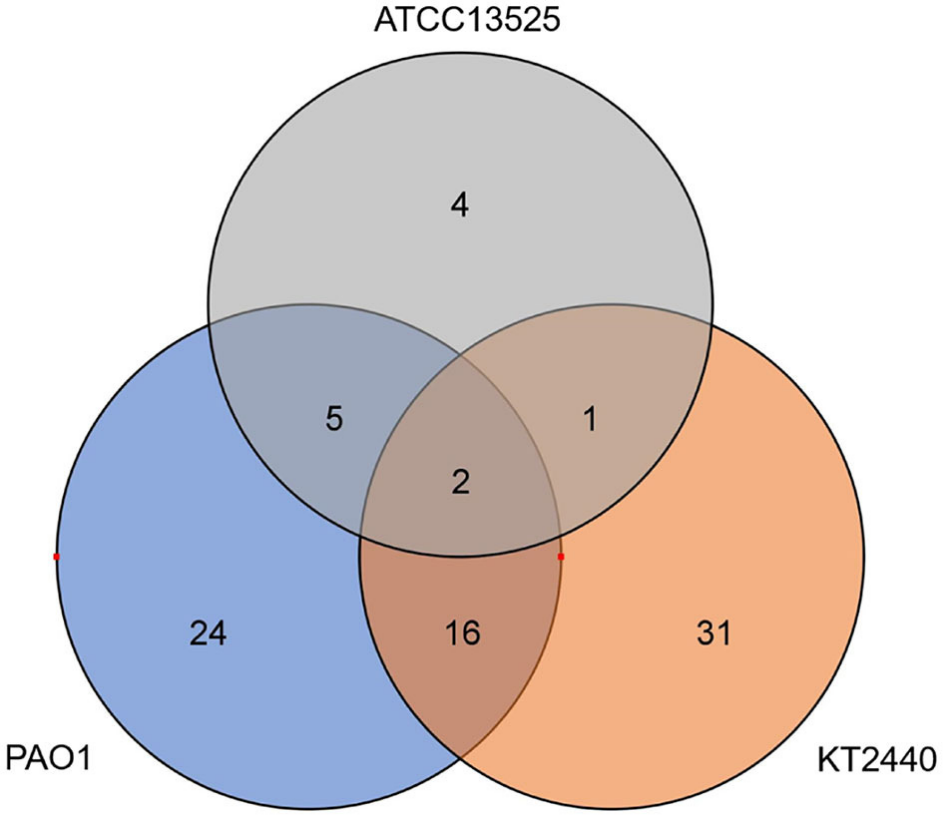
The quantity of metabolites commonly or strain-specifically regulated by *P. aeruginosa* PAO1, *P. putida* KT2440, and *P. fluorescence* ATCC 13525^T^.

Considering that large quantities of DEGs identified in the transcriptome analysis were genes associated with metabolism, we detected the concentrations of metabolites in these bacterial cells. Metabolic profiles of whole-cell extracts were obtained from zinc-treated and control samples exponentially cultured in CDM. Six replicates were performed for each sample. A total of 83 annotated metabolites were significantly changed, and approximately 76% of which were down-regulated. Only two metabolites were commonly regulated by the three *Pseudomonas* strains (pyroglutamic, 2-phosphoglycerate). The metabolite profiles of *P. aeruginosa* and *P. putida* shared more similarity, 18 metabolites were commonly regulated (Figure 3). However, after referring to the KEGG database, the metabolic pathways affected by the MICs of zinc imposed to the three *Pseudomonas* strains exhibited higher similarity (Supplementary Tables 8–10). Several metabolic intermediates associated with central carbon metabolisms (the Entner-Doudoroff pathway, tricarboxylic cycle, and pentose phosphate pathway) were significantly downregulated. The concentrations of citrate, phosphoenolpyruvate, and 2-phosphoglycerate in the zinc-treated *P. aeruginosa* PAO1 was only approximately 20% of that detected in the control samples. Similar phenomena were also observed in *P. putida* KT2440 and *P. fluorescens* ATCC 13525^T^. Compared with the *P. putida* cells without zinc treatment, the concentrations of citrate, succinate, and 2-phosphoglycerate were reduced by approximately 60.4, 72.6, and 78.2%, respectively. These observations are consistent with the RNA-seq data. The RPKM values of *mdh* (malate dehydrogenase) and *idh* (isocitrate dehydrogenase) in *P. putida* were only about 70% of that calculated in the cells without zinc treatment, and the transcription of *eda* (2-keto-3-deoxy-6-phosphogluconate aldolase) were downregulated about 1.7 and 1.5-fold in *P. putida* and *P. fluorescens*, respectively. The metabolites involved in amino acids metabolism were also reduced. For example, a wide range of amino acids (ornithine, Lys, Pro, Asp, Trp, and His) or intermediates involved in their biosynthesis were decreased in *P. putida*. And lower concentrations of metabolites involved in Arg, Glu, Lys, Cys, and Met metabolisms were observed in *P. aeruginosa*.

## Discussion

The genus *Pseudomonas* is one of the most diverse bacterial genera, comprising ~200 species isolated from sources ranging from vegetation to water and contaminated soil to animal clinical samples (Singh et al., 2018). With relatively large genomes (typically between 6 and 7 Mbp), many of these species have evolved delicate gene regulation systems that allow them to thrive under various environmental stresses (Jensen et al., 2004; Balasubramanian and Mathee, 2009). By combining the transcriptomic and metabolic data obtained in the present study, an overview of the global effects of zinc on three representative *Pseudomonas* strains was provided.

*P. aeruginosa* PAO1 was originally isolated from a human wound and widely studied as a model opportunistic human pathogen (Stover et al., 2000). Soil-originating *P. putida* KT2440 is the best-characterized strain from this species. It is used worldwide as the primary strain for genetic and physiological studies (Regenhardt et al., 2002). High genetic diversity was observed among bacteria classified as *P. fluorescens*, which can be further clustered into three smaller taxonomic subclades (Scales et al., 2015). Strains classified into one subclade shared a higher amount of conserved genes than the species-complex as a whole. Comparative genomic analysis showed a high level of genome similarity between *P. putida* KT2440 and *P. aeruginosa* PAO1, about 85% of the CDS are shared (Jensen et al., 2004). Although *P. fluorescens* ATCC 13525^T^ behaved as an out-group amongst the three strains, our bioinformatic analysis indicated that there is still 29% CDS are shared between P. *fluorescens* ATCC 13525^T^ and *P. putida* KT2440, and 33% CDS are shared between *P. fluorescens* ATCC 13525^T^ and *P. aeruginosa* PAO1. By contrast, the transcriptomic features of the three strains in response to the MICs of zinc exhibited significant divergence. Only 4 orthologs were commonly regulated, and all these orthologs were involved in metal efflux. The exclusion of cytoplasmic ions by membrane efflux pumps has been recognized as an important strategy for prokaryotic cells to withstand metal toxicity (Nies, 2007). However, this process is not energy-efficient, and damages can happen before these efflux systems are fully expressed. Reducing the entry of excess metal ions can more effectively prevent the generation of metal-induced cytotoxicity. Based on our data, the three strains may use different way to preclude the entry of excess zinc ions under high zinc conditions. *P. aeruginosa* PAO1 significantly decreased the secretion of siderophore, which was also found to complex with zinc, nickel, cobalt and magnesium as well as iron (Sasirekha and Srividya, 2016; Shi et al., 2017). *P. putida* universally reduced the expression of membrane transporters. *P. fluorescens* ATCC 13525^T^ has the lowest MIC value of zinc, and a relatively small number of genes were transcriptionally changed. Among these genes, T6SS gene cluster was remarkably downregulated, whose analogs in *B. thailandensis* play an important role in zinc uptake under oxidative stresses (Si et al., 2017). In addition to the change in zinc transport, it interesting to note the side effect caused by zinc ions. The increased alginate synthesis can facilitate bacterial adherence, biofilm formation, neutralize oxygen radicals, and act as a barrier to phagocytosis (Sautter et al., 2012). Coupled with the upregulated antibiotic efflux systems, the scavenging of zinc-exposed *P. aeruginosa* can be more difficult. *P. fluorescens* has been studied extensively as a plant growth promoter that synthesizes toxic metabolites against phytopathogenic microorganisms, and enhancing nutrient availability in soil (Loper et al., 2012). The downregulated transcription of T6SS can reduce their competence to occupy phyllosphere and rhizosphere, which in turn, decreases the effectiveness of *P. fluorescens* to be used as a biocontrol agent.

As transcriptome analysis showed that large quantities of DEGs identified in *P. aeruginosa, P. putida*, and *P. fluorescens* can be categorized into the metabolism KEGG group, Hence, different metabolic intermediates were measured using UPLC-MS/MS analysis to further explore the mechanisms underlying the effects of zinc exposure on the metabolic pathway. Two classes of metabolites were significantly decreased, including central carbon metabolism and amino acids synthesis, which may be one of the important reason why the growth of these bacterial cells stopped. Dipeptide is another group of metabolites that identified at lower concentrations after zinc treatment, especially in *P. aeruginosa* (Pro-Arg, and Ile-Leu) and *P. putida* (Pro-Val, Val-Ala, and Ile-Ala). High percentages of branched amino acids were observed, indicating that these dipeptides were not generated by protein degradation. The changes of Ile/Leu/Val containing short peptides were also observed by Jousse et al. (2017). Their metabolomics data revealed that the concentrations of Ala-Leu, Leu-Leu, Ala-Arg, Pro-Phe, Pro-Ile, Leu-Phe, and Val-Val were significantly decreased in cold-shocked *P. syringae*. Beside the downregulated compounds discussed above, some metabolites were observed at high concentrations, such as nucleoside/base (adenosine, deoxycytidine, uracil, cytosine) and proline. Proline has been reported to play important roles in protecting protein denaturation and stabilizing protein synthesis, the upregulation of which commonly occurs in cells under heavy metal stresses (Alia and Saradhi, 1991; Choudhary et al., 2007). Because energy generation and central carbon metabolism were severely affected and the upregulation of exonuclease in *P. aeruginosa* PAO1 (PA4937) and *P. putida* KT2440 (PP_0034, PP_0353) was observed (<2 fold), the presence of high concentrations of nucleotide and base may be produced by the recycling of denatured nucleic acid.

In this study, we compared the zinc-induced cellular responses of three prominent *Pseudomonas* strains from different phylogenetic groups. The analysis of the obtained transcriptomic profiles showed that 1,120 genes transcriptionally changed by more than twofold. The exclusion of cytoplasmic ions and reducing the unexpected intrusion of excess zinc are important for *P. aeruginosa, P. putida*, and *P. fluorescens* to withstand external zinc stress. Metabolic analysis showed that low concentrations of the metabolic intermediates involved in central carbon metabolism and amino acid biosynthesis were identified. Dipeptides that contained branched-chain amino acids may play important roles during their zinc adaptation.

## Data Availability Statement

The RNA-seq datasets for this study can be found in the NCBI short read archive database under the bioproject accession number PRJNA606809.

## Author Contributions

LL and PL conceived the experiment. LL, WL, and JC performed the experiment. LL and PL wrote the manuscript. All authors contributed to the article and approved the submitted version.

## Conflict of Interest

The authors declare that the research was conducted in the absence of any commercial or financial relationships that could be construed as a potential conflict of interest.
