# Supplementary material for: Determining the Different Mechanisms Used by Pseudomonas Species to Cope With Minimal Inhibitory Concentrations of Zinc via Comparative Transcriptomic Analyses
Source: Front Microbiol. 2020 Dec 3;11:573857. doi: 10.3389/fmicb.2020.573857 (PMC7744410; doi:10.3389/fmicb.2020.573857)
Supplement: Supplementary file 1 [file Data_Sheet_1.docx]

Supplementary Material

# Supplementary Tables

Table S1 Primer pairs used in this study.

| Strain | Gene | Primer pairs | Product size |
| --- | --- | --- | --- |
| *P. aeruginosa* PAO1 | *nuoA* | TGAACTTGCAGCTCATCACTG | 131 |
|  |  | CGAAGGGTTCGTTCTTGC |  |
|  | *algU* | CGATGTGACCGCAGAGGA | 133 |
|  |  | CTCGGGCAACTGCTGGAT |  |
|  | *pvdA* | TCATCGGTGTCGGCTTCG | 115 |
|  |  | CGGTAGTCGCCCTGCTTGT |  |
|  | *rplS* | GCGATACCGTGATCGTCC | 171 |
|  |  | TCGGGCTGTAGGTCTGGAA |  |
|  | *atpE* | AAATGGTTCCGATGCTGC | 120 |
|  |  | TTTGACCAACGAAGGGATT |  |
|  | *dnaE* | AAGAAGAAGCCGAGGAAATG | 139 |
|  |  | CTACGGCTTCAACAAGTCGC |  |
| *P. putida* KT2440 | *nikA* | TGGGCTGGCAATCAAGGA | 138 |
|  |  | TGCGAGATGGGCAGGTAGA |  |
|  | *rpmH* | ACTTTCCAACCAAGCACCATC | 109 |
|  |  | TACGGCCTTTGGCACGAC |  |
|  | *gtsB* | GTGGGTGGCGAGCAAGAA | 133 |
|  |  | CAGGTAAATGGTGCGAATGAA |  |
|  | *livM* | GGCTACGTGGGCTTCTATGC | 187 |
|  |  | CACCGAAACCGAGGGTCA |  |
|  | *fruK* | ATGGCCAAGATCCTCAC | 134 |
|  |  | GTGAGGATCTTGGCCAT |  |
|  | *gltA* | CACCGTTGGTCCTGATGTAA | 172 |
|  |  | CTGTTCGGCGAGTTGCTC |  |
| *P. fluorescens* ATCC13525 | *glpK* | ACAAAGCCGTCAAATCCAAA | 199 |
|  |  | TAGACGCCGTTGCTGTCC |  |
|  | *emrB* | CCGTTGTACCCGATGACCC | 132 |
|  |  | GATCCAGCCGCCGAGAAT |  |
|  | *tssJ* | CCGCCGACAAAGACCTCA | 100 |
|  |  | CCGGTGTAGGCACTGAACG |  |
|  | *hcp* | GCCAGGTCGAGTACATGGTG | 116 |
|  |  | TTGGGCGAAGTTCAGGGT |  |
|  | *znuB* | GCGATGAAGATTGTCGGCGTAT | 168 |
|  |  | CGGCGTGTCCTTGAACCAG |  |
|  | *zntB* | ACCCGTTACCTCGAAGAGC | 173 |
|  |  | CCCACATTTATACCCAGCAGA |  |

Table S2 DEGs mutually regulated by *P. aeruginosa* PAO1 and *P. putida* KT2440

| ORF | | Gene name | | Fold change^a^ | | Annotation |
| --- | --- | --- | --- | --- | --- | --- |
| PAO1 | KT2440 | PAO1 | KT2440 | PAO1 | KT2440 |  |
| Metal homeostasis | | |  |  |  |  |
| PA2520 | PP_0043 | *czcA* | *czcA1* | 77.01 | 38.32 | RND divalent metal cation efflux transporter |
| PA2521 | PP_0044 | *czcB* | *czcB1* | 71.5 | 29.04 | RND divalent metal cation efflux membrane fusion protein |
| PA2522 | PP_0045 | *czcC* | *czcC1* | 62.44 | 33.59 | outer membrane protein precursor |
| PA2523 | PP_1438 | *czcR* | *czcR2* | 94.44 | 30.48 | two-component response regulator |
| PA2524 | PP_1437 | *czcS* | *czcS2* | 39.6 | 14.22 | sensor histidine kinase |
| PA3689 | PP_5138 | *cadR* | *cadR* | 22.89 | 64.28 | Cd/Pb-response regulator |
| PA3690 | PP_5139 | *cadA* | *cadA3* | 65.19 | 101.83 | metal-transporting P-type ATPase |
| Membrane structure and channels | | | |  |  |  |
| PA0286 | PP_0217 | *desA* | *desA* | 5.72 | 2.79 | delta-9 fatty acid desaturase |
| PA0846 | PP_0785 | *cysZ* | *cysZ* | 2.27 | 2.77 | sulfate uptake protein |
| PA0958 | PP_1206 | *oprD* | *oprD* | 0.31 | 0.23 | basic amino acid, basic peptide outer membrane porin |
| PA2262 | PP_3377 | *–* | *kguT* | 0.34 | 0.28 | probable 2-ketogluconate transporter |
| PA2525 | PP_3582 | *ompB* | *–* | 6.07 | 2.16 | RND transporter outer membrane protein |
| PA2526 | PP_3583 | *–* | *mdtC* | 3.16 | 2.17 | RND efflux transporter |
| PA2760 | PP_0268 | *–* | *oprQ* | 0.29 | 4.5 | outer membrane porin |
| PA2857 | PP_2317 | *–* | *ybbA* | 2.16 | 2.62 | ATP-binding component of ABC transporter |
| PA2987 | PP_2155 | *–* | *lolD* | 2.2 | 2.11 | lipoprotein localization protein |
| PA2988 | PP_2154 | *–* | *lolC* | 2.1 | 2.06 | lipoprotein localization protein |
| PA3234 | PP_1743 | *–* | *actP1* | 2.66 | 2.2 | probable sodium:solute symporter |
| PA3603 | PP_1636 | *dgkA* | *dgkA1* | 10.88 | 5.03 | diacylglycerol kinase |
| PA4378 | PP_0904 | *warB* | *–* | 22.49 | 3.66 | InaA protein |
| PA4379 | PP_0903 | *warA* | *–* | 26.01 | 3.81 |  |
| PA4819 | PP_0034 | *–* | *–* | 28.5 | 125.37 | bactoprenol glycosyl-transferase |
| PA5200 | PP_0246 | *amgR* | *ompR* | 9.36 | 2.58 | two-component system DNA-binding response regulator |
| PA5530 | PP_1400 | *–* | *kgtP* | 3.83 | 0.28 | alpha-ketoglutarate permease |
| Protein folding and degradation | | | |  |  |  |
| PA0779 | PP_1443 | *asrA* | *lon1* | 11.61 | 3.84 | ATP-dependent protease |
| PA1596 | PP_4179 | *htpG* | *htpG* | 4.6 | 6.02 | chaperone protein |
| PA1803 | PP_2302 | *lon* | *lon2* | 3.23 | 2.71 | ATP-dependent protease |
| PA2830 | PP_1871 | *htpX* | *htpX* | 29.61 | 3.12 | heat shock protein |
| PA3126 | PP_1982 | *ibpA* | *ibpA* | 12.34 | 4.86 | heat shock protein |
| PA3811 | PP_0845 | *hscB* | *hscB* | 2.53 | 2.16 | heat shock protein HscB |
| PA4385 | PP_1361 | *groEL* | *groEL* | 3.97 | 6.02 | chaperonin |
| PA4386 | PP_1360 | *groES* | *groES* | 4.72 | 5.5 | chaperonin |
| PA4542 | PP_0625 | *clpB* | *clpB* | 10 | 2.5 | ATP-dependent protease |
| PA4760 | PP_4726 | *dnaJ* | *dnaJ* | 4.53 | 3.61 | heat shock protein |
| PA4761 | PP_4727 | *dnaK* | *dnaK* | 7.35 | 5.31 | heat shock protein |
| PA4762 | PP_4728 | *grpE* | *grpE* | 3.12 | 5.28 | heat shock protein |
| PA5053 | PP_5000 | *hslV* | *hslV* | 7.9 | 8.57 | heat shock protein |
| PA5054 | PP_5001 | *hslU* | *hslU* | 7.02 | 6.5 | heat shock protein |
| Basic metabolism | |  |  |  |  |  |
| PA0887 | PP_4487 | *acsA* | *acsA1* | 2.66 | 5.82 | acetyl-coenzyme A synthetase |
| PA1317 | PP_0812 | *cyoA* | *cyoA* | 2.1 | 3.23 | cytochrome o ubiquinol oxidase subunit II |
| PA1318 | PP_0813 | *cyoB* | *cyoB* | 2.11 | 3.12 | cytochrome o ubiquinol oxidase subunit I |
| PA1319 | PP_0814 | *cyoC* | *cyoC* | 2.11 | 3.32 | cytochrome o ubiquinol oxidase subunit III |
| PA1320 | PP_0815 | *cyoD* | *cyoD* | 2.14 | 3.36 | cytochrome o ubiquinol oxidase subunit IV |
| PA1321 | PP_0816 | *cyoE* | *cyoE* | 2.14 | 3.01 | cytochrome o ubiquinol oxidase subunit V |
| PA2624 | PP_4012 | *idh* | *idh* | 2.73 | 2.36 | isocitrate dehydrogenase |
| PA3812 | PP_0844 | *iscA* | *iscA* | 2.66 | 2.45 | probable iron-binding protein |
| PA3814 | PP_0842 | *iscS* | *iscS1* | 2.75 | 2.43 | L-cysteine desulfurase |
| PA3815 | PP_0841 | *iscR* | *iscR* | 2.54 | 2.23 | DNA-binding transcriptional regulator |
| Others |  |  |  |  |  |  |
| PA0393 | PP_5095 | *proC* | *prol* | 3.08 | 2.2 | pyrroline-5-carboxylate reductase |
| PA2261 | PP_3378 | *–* | *kguT* | 0.36 | 0.28 | probable 2-ketogluconate kinase |
| PA2263 | PP_3376 | *–* | *ptxD* | 0.31 | 0.37 | probable 2-hydroxyacid dehydrogenase |
| PA2414 | PP_3613 | *–* | *–* | 3.61 | 2.13 | L-sorbosone dehydrogenase |
| PA2561 | PP_2120 | *ctpH* | *ctpH* | 2.01 | 2.43 | chemotactic transducer |
| PA2631 | PP_4018 | *–* | *–* | 2.58 | 3.2 | acetyl transferase |
| PA3710 | PP_0056 | *–* | *betA1* | 2.95 | 2.3 | probable GMC-type oxidoreductase |
| PA4356 | PP_0920 | *xenB* | *xenB* | 2.78 | 3.94 | xenobiotic reductase |
| PA4759 | PP_4725 | *dapB* | *dapB* | 3.64 | 3.39 | dihydrodipicolinate reductase |

^a^ Mean values of three bioreplicates

Table S3 DEGs mutually regulated by *P. aeruginosa* PAO1 and *P. fluorescens* ATCC13525

| ORF | | Gene name | | Fold change^a^ | | Annotation |
| --- | --- | --- | --- | --- | --- | --- |
| PAO1 | ATCC13525 | PAO1 | ATCC13525 | PAO1 | ATCC13525 |  |
| PA0434 | RS23050 |  |  | 0.39 | 0.09 | Ton-dependent receptor |
| PA0506 | RS07420 |  |  | 2.45 | 4.3 | probable acyl-CoA dehydrogenase |
| PA2402 | RS27315 |  |  | 0.33 | 0.253 | non-ribosomal peptide synthetase |
| PA2523 | RS13885 | *czcR* |  | 94.44 | 4.63 | two-component response regulator |
| PA2524 | RS13880 | *czcS* |  | 39.6 | 5 | histdine kinase |
| PA3556 | RS29670 | *arnT* |  | 3.88 | 2.8 | inner membrane L-Ara4N transferase |
| PA3689 | RS08415 | *cadR* |  | 22.89 | 4.15 | Cd/Pb-responsive regulator |
| PA3690 | RS08410 | *cadA* |  | 65.19 | 18.11 | metal-transporting P-type ATPase |
| PA3885 | RS10450 |  |  | 11.11 | 2.892 | protein tyrosine phosphatase TpbA |
| PA3972 | RS06160 |  |  | 3.77 | 2.11 | probable acyl-CoA dehydrogenase |
| PA5158 | RS29025 |  |  | 0.35 | 2.061 | outer membrane protein precursor |
| PA5216 | RS21745 |  |  | 3.12 | 2.17 | probable permease of iron transporter |
| PA5436 | RS09175 |  |  | 0.23 | 2.18 | biotin carboxylase subunit |

^a^ Mean values of three bioreplicates

Table S4 DEGs mutually regulated by *P. putida* KT2440 and *P. fluorescens* ATCC13525

| ORF |  | Gene name | | Fold change^a^ | | Annotation | |
| --- | --- | --- | --- | --- | --- | --- | --- |
| KT2440 | ATCC13525 | KT2440 | ATCC13525 | KT2440 | ATCC13525 | |  |
| PP_0411 | RS21740 |  |  | 0.11 | 2.06 | polyamine ABC trnasporter | |
| PP_1263 | RS19005 |  |  | 35.94 | 2.06 | fusaric acid resistance protein | |
| PP_1437 | RS13880 | *czcS2* |  | 14.22 | 5 | heavy metal sensor histidine kinase | |
| PP_1438 | RS13885 | *czcR2* |  | 30.48 | 4.63 | response regulator | |
| PP_2453 | RS29965 |  |  | 5.88 | 2.44 | glutaminase-asparaginase | |
| PP_5138 | RS08405 | *cadR* |  | 64.28 | 8.27 | Cd/Pb-response regulator | |
| PP_5139 | RS08410 | *cadA3* |  | 101.83 | 18.81 | cadmium translocating P-type ATPase | |

^a^ Mean values of three bioreplicates

Table S5 DEGs specifically regulated by *P. aeruginosa* PAO1^a^.

| ORF | Gene | Fold change^b^ | P_adjust_ | Annotation |
| --- | --- | --- | --- | --- |
| **PA0035** | ***trpA*** | **0.155** | **5.4E-08** | **tryptophan synthase subunit alpha** |
| **PA0036** | ***trpB*** | **0.448** | **0.02028** | **tryptophan synthase subunit beta** |
| PA0059 | *osmC* | 3.887 | 2.2E-05 | osmotically inducible protein OsmC |
| PA0102 | *-* | 17.516 | 1E-24 | carbonic anhydrase |
| PA0103 | *-* | 15.605 | 2.9E-21 | sulfate transporter |
| PA0183 | *atsA* | 0.377 | 0.00458 | arylsulfatase |
| **PA0198** | ***exbB1*** | **0.236** | **1.9E-05** | **transporter ExbB** |
| **PA0199** | ***exbD1*** | **0.148** | **1.5E-07** | **biopolymer transport protein ExbD** |
| PA0208 | *mdcA* | 3.45 | 0.00014 | malonate decarboxylase subunit alpha |
| PA0232 | *pcaC* | 2.728 | 0.00661 | 4-carboxymuconolactone decarboxylase |
| PA0291 | *oprE* | 0.284 | 0.00023 | anaerobically-induced outer membrane porin OprE |
| PA0382 | *micA* | 0.437 | 0.04013 | tRNA (guanine-N(7)-)-methyltransferase |
| **PA0424** | ***mexR*** | **2.722** | **0.00176** | **multidrug resistance operon repressor MexR** |
| **PA0425** | ***mexA*** | **4.461** | **8.5E-06** | **multidrug resistance protein MexA** |
| **PA0426** | ***mexB*** | **4.452** | **1.7E-05** | **multidrug resistance protein MexB** |
| **PA0427** | ***oprM*** | **3.318** | **0.00066** | **outer membrane protein OprM** |
| **PA0518** | ***nirM*** | **3.405** | **0.00618** | **cytochrome C-551** |
| **PA0519** | ***nirS*** | **4.078** | **2.5E-06** | **nitrite reductase** |
| **PA0520** | ***nirQ*** | **2.771** | **0.00338** | **denitrification regulatory protein NirQ** |
| **PA0523** | ***norC*** | **8.402** | **1.1E-13** | **nitric oxide reductase subunit C** |
| **PA0524** | ***norB*** | **8.858** | **4.3E-14** | **nitric oxide reductase subunit B** |
| **PA0525** | ***-*** | **3.313** | **0.0001** | **denitrification protein NorD** |
| PA0730 | *-* | 0.325 | 0.00425 | (R)-3-hydroxydecanoyl-ACP:CoA transacylase |
| **PA0762** | ***algU*** | **4.241** | **0.00013** | **RNA polymerase sigma factor AlgU** |
| **PA0763** | ***mucA*** | **6.057** | **1.6E-07** | **sigma factor AlgU negative regulator MucA** |
| **PA0764** | ***mucB*** | **4.581** | **1.4E-05** | **sigma factor AlgU regulator MucB** |
| **PA0765** | ***mucC*** | **3.594** | **0.00027** | **positive regulator for alginate biosynthesis MucC** |
| PA0778 | *icp* | 2.717 | 0.00675 | inhibitor of cysteine peptidase |
| PA0780 | *pruR* | 2.339 | 0.01841 | proline utilization regulator |
| PA0807 | *ampDh3* | 2.166 | 0.04369 | protein AmpDh3 |
| PA0809 | *-* | 2.391 | 0.01203 | divalent metal cation transporter MntH |
| PA0840 | *-* | 2.216 | 0.02974 | oxidoreductase |
| PA0852 | *cbpD* | 0.428 | 0.04116 | chitin-binding protein CbpD |
| **PA0853** | ***-*** | **2.434** | **0.01852** | **oxidoreductase** |
| **PA0854** | ***fumC2*** | **2.31** | **0.02295** | **fumarate hydratase** |
| PA0870 | *phhC* | 2.586 | 0.00955 | aromatic amino acid aminotransferase |
| PA0905 | *rsmA* | 3.601 | 0.00143 | carbon storage regulator |
| PA0918 | *-* | 3.271 | 0.00148 | cytochrome b561 |
| PA0932 | *cysM* | 2.398 | 0.03569 | cysteine synthase B |
| **PA1027** | ***-*** | **13.656** | **8E-22** | **aldehyde dehydrogenase** |
| **PA1028** | ***-*** | **7.507** | **3E-13** | **oxidoreductase** |
| PA1054 | *-* | 2.547 | 0.00458 | monovalent cation/H+ antiporter subunit A |
| **PA1092** | ***fliC*** | **3.306** | **0.00117** | **B-type flagellin** |
| **PA1094** | ***fliD*** | **2.341** | **0.04523** | **B-type flagellar hook-associated protein** |
| **PA1095** | ***-*** | **2.502** | **0.03376** | **B-type flagellar protein FliS** |
| PA1156 | *nrdA* | 0.371 | 0.0083 | ribonucleotide-diphosphate reductase subunit alpha |
| PA1178 | *oprH* | 0.386 | 0.0241 | PhoP/Q and low Mg2+ inducible outer membrane protein |
| PA1297 | *-* | 2.747 | 0.00299 | metal transporter |
| PA1306 | *-* | 2.419 | 0.01807 | HIT family protein |
| **PA1437** | ***-*** | **3.226** | **0.0004** | **two-component response regulator** |
| **PA1438** | ***-*** | **2.728** | **0.00247** | **two-component sensor** |
| PA1549 | *-* | 2.182 | 0.03442 | cation-transporting P-type ATPase |
| PA1551 | *-* | 3.48 | 0.0001 | ferredoxin |
| PA1561 | *aer* | 2.828 | 0.00142 | aerotaxis receptor Aer |
| PA1562 | *acnA* | 3.628 | 3.9E-05 | aconitate hydratase |
| PA1617 | *-* | 2.122 | 0.04517 | AMP-binding protein |
| PA1742 | *-* | 4.705 | 3.7E-06 | amidotransferase |
| PA1756 | *cysH* | 2.128 | 0.04961 | phosphoadenosine phosphosulfate reductase |
| PA1880 | *-* | 2.331 | 0.01515 | oxidoreductase |
| **PA1899** | ***phzA2*** | **0.082** | **1.7E-14** | **phenazine biosynthesis protein PhzA** |
| **PA1900** | ***phzB2*** | **0.196** | **3E-06** | **phenazine biosynthesis protein PhzB** |
| **PA1901** | ***phzC2*** | **0.38** | **0.02265** | **phenazine biosynthesis protein PhzC** |
| **PA1902** | ***phzD2*** | **0.423** | **0.04911** | **phenazine biosynthesis protein PhzD** |
| **PA1905** | ***phzG2*** | **0.407** | **0.027** | **pyridoxamine 5'-phosphate oxidase** |
| PA1973 | *pqqF* | 3.248 | 0.00094 | coenzyme PQQ synthesis protein F |
| PA2000 | *dhcB* | 0.404 | 0.02547 | dehydrocarnitine CoA transferase subunit B |
| PA2008 | *fahA* | 2.891 | 0.00106 | fumarylacetoacetase |
| **PA2018** | ***-*** | **5.877** | **3.8E-09** | **multidrug efflux protein** |
| **PA2019** | ***-*** | **6.135** | **3.8E-09** | **multidrug efflux lipoprotein** |
| PA2020 | *-* | 3.914 | 1.2E-05 | transcriptional regulator |
| PA2023 | *galU* | 2.792 | 0.01017 | UTP-glucose-1-phosphate uridylyltransferase |
| PA2024 | *-* | 3.486 | 0.00051 | ring-cleaving dioxygenase |
| PA2047 | *-* | 2.855 | 0.00166 | transcriptional regulator |
| PA2051 | *-* | 0.441 | 0.03261 | transmembrane sensor |
| PA2086 | *-* | 0.414 | 0.01877 | epoxide hydrolase |
| PA2092 | *-* | 0.435 | 0.02379 | major facilitator superfamily transporter |
| **PA2093** | ***-*** | **0.379** | **0.01771** | **RNA polymerase sigma factor** |
| **PA2094** | ***-*** | **0.437** | **0.03825** | **transmembrane sensor** |
| PA2144 | *glgP* | 2.654 | 0.00334 | glycogen phosphorylase |
| **PA2151** | ***-*** | **2.85** | **0.00156** | **alpha-1,4-glucan:maltose-1-phosphate maltosyltransferase** |
| **PA2152** | ***-*** | **2.923** | **0.00091** | **trehalose synthase** |
| PA2160 | *-* | 3.619 | 2E-05 | glycosyl hydrolase |
| PA2163 | *-* | 2.352 | 0.02075 | 4-alpha-glucanotransferase |
| PA2165 | *-* | 2.892 | 0.00117 | glycogen synthase |
| PA2272 | *pbpC* | 0.353 | 0.00225 | penicillin-binding protein 3A |
| PA2312 | *-* | 2.507 | 0.016 | transcriptional regulator |
| PA2344 | *mtlZ* | 2.51 | 0.01259 | fructokinase |
| PA2359 | *-* | 3.08 | 0.00072 | transcriptional regulator |
| PA2379 | *-* | 3.027 | 0.00199 | oxidoreductase |
| **PA2385** | ***pvdQ*** | **0.338** | **0.00432** | **acyl-homoserine lactone acylase PvdQ** |
| **PA2386** | ***pvdA*** | **0.29** | **0.00088** | **L-ornithine N5-oxygenase** |
| **PA2392** | ***pvdP*** | **0.365** | **0.00663** | **pyoverdine biosynthesis protein PvdP** |
| **PA2393** | ***-*** | **0.302** | **0.00101** | **dipeptidase** |
| **PA2394** | ***pvdN*** | **0.261** | **9.9E-05** | **pyoverdine biosynthesis protein PvdN** |
| **PA2395** | ***pvdO*** | **0.24** | **6.9E-05** | **pyoverdine biosynthesis protein PvdO** |
| **PA2396** | ***pvdF*** | **0.195** | **4.1E-06** | **pyoverdine synthetase F** |
| **PA2397** | ***pvdE*** | **0.366** | **0.00681** | **pyoverdine biosynthesis protein PvdE** |
| **PA2398** | ***fpvA*** | **0.337** | **0.00442** | **ferripyoverdine receptor** |
| **PA2399** | ***pvdD*** | **0.34** | **0.00677** | **pyoverdine synthetase D** |
| **PA2400** | ***pvdJ*** | **0.292** | **0.00119** | **pyoverdine biosynthesis protein PvdJ** |
| **PA2407** | ***-*** | **3.965** | **4.5E-06** | **adhesion protein** |
| **PA2408** | ***-*** | **4.131** | **3E-06** | **ABC transporter ATP-binding protein** |
| **PA2409** | ***-*** | **3.805** | **1.2E-05** | **ABC transporter permease** |
| **PA2424** | ***pvdL*** | **0.343** | **0.00598** | **peptide synthase** |
| **PA2426** | ***pvdS*** | **0.194** | **8.6E-07** | **extracytoplasmic-function sigma-70 factor** |
| PA2505 | *opdT* | 8.242 | 1.7E-11 | tyrosine porin OpdT |
| PA2507 | *catA* | 2.788 | 0.03146 | catechol 1%2C2-dioxygenase |
| PA2527 | *-* | 2.81 | 0.00184 | resistance-nodulation-cell division (RND) efflux transporter |
| PA2531 | *-* | 0.351 | 0.00208 | aminotransferase |
| PA2620 | *clpA* | 2.536 | 0.02806 | ATP-binding protease component ClpA |
| **PA2637** | ***nuoA*** | **2.923** | **0.01391** | **NADH-quinone oxidoreductase subunit A** |
| **PA2642** | ***nuoG*** | **2.326** | **0.01963** | **NADH-quinone oxidoreductase subunit G** |
| **PA2643** | ***nuoH*** | **2.519** | **0.01251** | **NADH-quinone oxidoreductase subunit H** |
| **PA2644** | ***nuoI*** | **2.339** | **0.03048** | **NADH-quinone oxidoreductase subunit I** |
| **PA2645** | ***nuoJ*** | **2.35** | **0.03695** | **NADH-quinone oxidoreductase subunit J** |
| **PA2647** | ***nuoL*** | **2.342** | **0.01328** | **NADH-quinone oxidoreductase subunit L** |
| **PA2648** | ***nuoM*** | **2.256** | **0.02788** | **NADH-quinone oxidoreductase subunit M** |
| **PA2649** | ***nuoN*** | **2.098** | **0.04671** | **NADH-quinone oxidoreductase subunit N** |
| **PA2656** | ***-*** | **12.721** | **9.7E-20** | **two-component sensor** |
| **PA2657** | ***-*** | **19.816** | **8.4E-28** | **two-component response regulator** |
| PA2664 | *fhp* | 3.157 | 0.00026 | flavohemoprotein |
| PA2788 | *-* | 2.422 | 0.02268 | chemotaxis transducer |
| PA2806 | *-* | 2.476 | 0.00929 | 7-cyano-7-deazaguanine reductase |
| PA2808 | *ptrA* | 1255.141 | 6E-121 | repressor PtrA |
| PA2815 | *-* | 3.459 | 0.00026 | acyl-CoA dehydrogenase |
| PA2825 | *ospR* | 4.265 | 6.4E-06 | transcriptional regulator |
| PA2826 | *-* | 6.105 | 3.9E-09 | glutathione peroxidase |
| PA2859 | *greB* | 2.46 | 0.01963 | transcription elongation factor GreB |
| PA2896 | *-* | 4.004 | 4.4E-06 | RNA polymerase sigma factor |
| PA2920 | *-* | 2.161 | 0.04264 | chemotaxis transducer |
| PA2939 | *-* | 0.378 | 0.01498 | aminopeptidase |
| PA2961 | *holB* | 2.313 | 0.03146 | DNA polymerase III subunit delta' |
| PA3023 | *-* | 2.554 | 0.00737 | lipid kinase |
| PA3206 | *-* | 6.231 | 3.3E-10 | two-component sensor |
| PA3272 | *-* | 2.225 | 0.02028 | ATP-dependent DNA helicase |
| PA3327 | *-* | 0.47 | 0.04889 | non-ribosomal peptide synthetase |
| PA3351 | *flgM* | 3.651 | 0.00101 | protein FlgM anti-sigma factor |
| **PA3391** | ***nosR*** | **2.788** | **0.00625** | **regulatory protein NosR** |
| **PA3392** | ***nosZ*** | **2.615** | **0.00497** | **nitrous-oxide reductase** |
| PA3405 | *hasE* | 0.457 | 0.04698 | metalloprotease secretion protein |
| PA3408 | *hasR* | 0.408 | 0.02213 | heme uptake outer membrane receptor HasR |
| **PA3415** | ***-*** | **2.393** | **0.02743** | **branched-chain alpha-keto acid dehydrogenase subunit E2** |
| **PA3416** | ***-*** | **2.722** | **0.00394** | **pyruvate dehydrogenase E1 component subunit beta** |
| **PA3417** | ***-*** | **2.488** | **0.00927** | **pyruvate dehydrogenase E1 component subunit alpha** |
| **PA3418** | ***ldh*** | **2.326** | **0.03627** | **leucine dehydrogenase** |
| **PA3459** | ***-*** | **4.724** | **8.2E-08** | **glutamine amidotransferase** |
| **PA3460** | ***-*** | **3.771** | **7.9E-06** | **acetyltransferase** |
| **PA3552** | ***arnB*** | **3.227** | **0.00094** | **UDP-4-amino-4-deoxy-L-arabinose--oxoglutarate aminotransferase** |
| **PA3553** | ***arnC*** | **3.483** | **0.00026** | **undecaprenyl-phosphate 4-deoxy-4-formamido-L-arabinose transferase** |
| **PA3554** | ***arnA*** | **3.543** | **0.00038** | **bifunctional UDP-glucuronic acid decarboxylase/UDP-4-amino-4-deoxy-L-arabinose formyltransferase** |
| **PA3555** | ***arnD*** | **3.801** | **7.5E-05** | **4-deoxy-4-formamido-L-arabinose-phosphoundecaprenol deformylase ArnD** |
| **PA3557** | ***arnE*** | **3.635** | **0.00096** | **4-amino-4-deoxy-L-arabinose-phosphoundecaprenol flippase subunit ArnE** |
| **PA3558** | ***arnF*** | **3.174** | **0.00117** | **4-amino-4-deoxy-L-arabinose-phosphoundecaprenol flippase subunit ArnF** |
| **PA3559** | ***-*** | **3.982** | **4.9E-05** | **nucleotide sugar dehydrogenase** |
| PA3656 | *rpsB* | 0.404 | 0.02942 | 30S ribosomal protein S2 |
| PA3692 | *lptF* | 5.377 | 9.4E-07 | outer membrane porin F |
| PA3742 | *rplS* | 0.391 | 0.03866 | 50S ribosomal protein L19 |
| PA3795 | *-* | 2.282 | 0.02358 | oxidoreductase |
| PA3818 | *-* | 2.205 | 0.04889 | type III secretion system regulator SuhB |
| PA3891 | *-* | 2.223 | 0.02942 | ABC transporter ATP-binding protein |
| **PA3929** | ***cioB*** | **3.287** | **0.00133** | **cyanide insensitive terminal oxidase** |
| **PA3930** | ***cioA*** | **3.995** | **0.00011** | **cyanide insensitive terminal oxidase** |
| PA4061 | *-* | 2.367 | 0.02265 | thioredoxin |
| PA4168 | *fpvB* | 0.236 | 1.4E-05 | second ferric pyoverdine receptor FpvB |
| PA4175 | *piv* | 0.38 | 0.01237 | endopeptidase IV |
| PA4209 | *phzM* | 4.004 | 4.5E-06 | phenazine-specific methyltransferase |
| PA4213 | *phzD1* | 0.423 | 0.04911 | phenazine biosynthesis protein PhzD |
| **PA4259** | ***rpsS*** | **0.291** | **0.00109** | **30S ribosomal protein S19** |
| **PA4261** | ***rplW*** | **0.374** | **0.02353** | **50S ribosomal protein L23** |
| **PA4269** | ***rpoC*** | **0.38** | **0.02028** | **DNA-directed RNA polymerase subunit beta'** |
| **PA4270** | ***rpoB*** | **0.406** | **0.0345** | **DNA-directed RNA polymerase subunit beta** |
| PA4272 | *rplJ* | 0.409 | 0.03866 | 50S ribosomal protein L10 |
| PA4309 | *pctA* | 2.413 | 0.01457 | chemotactic transducer PctA |
| PA4344 | *-* | 2.304 | 0.02478 | hydrolase |
| **PA4358** | ***feoB*** | **6.05** | **1.6E-07** | **ferrous iron transporter B** |
| **PA4359** | ***feoA-*** | **6.07** | **8.5E-09** | **ferrous iron transporter A** |
| PA4370 | *icmP* | 0.337 | 0.00323 | insulin-cleaving metalloproteinase outer membrane protein |
| PA4381 | *-* | 2.709 | 0.00331 | two-component response regulator |
| PA4472 | *pmbA* | 2.529 | 0.01519 | PmbA protein |
| PA4514 | *-* | 0.352 | 0.00409 | iron transport outer membrane receptor |
| PA4563 | *rpsT* | 0.309 | 0.0048 | 30S ribosomal protein S20 |
| **PA4567** | ***rpmA*** | **0.354** | **0.04098** | **50S ribosomal protein L27** |
| **PA4568** | ***rplU*** | **0.381** | **0.01999** | **50S ribosomal protein L21** |
| **PA4661** | ***pagL*** | **3.591** | **0.00076** | **lipid A 3-O-deacylase** |
| PA4671 | *-* | 0.333 | 0.0048 | 50S ribosomal protein L25/general stress protein Ctc |
| PA4710 | *phuR* | 0.26 | 9.7E-05 | heme/hemoglobin uptake outer membrane receptor PhuR |
| **PA4749** | ***glmM*** | **3.021** | **0.00368** | **phosphoglucosamine mutase** |
| **PA4750** | ***folP*** | **4.222** | **3.2E-05** | **dihydropteroate synthase** |
| **PA4751** | ***ftsH*** | **5.208** | **2.6E-06** | **cell division protein FtsH** |
| **PA4752** | ***ftsJ*** | **2.815** | **0.00371** | **cell division protein FtsJ** |
| PA4763 | *recN* | 2.133 | 0.03189 | DNA repair protein RecN |
| **PA4776** | ***pmrA*** | **3.386** | **0.00085** | **two-component regulator system response regulator PmrA** |
| **PA4777** | ***pmrB*** | **3.367** | **0.00043** | **two-component regulator system signal sensor kinase PmrB** |
| PA4843 | *-* | 2.329 | 0.04961 | two-component response regulator |
| **PA4853** | ***fis*** | **0.302** | **0.00675** | **Fis family transcriptional regulator** |
| **PA4854** | ***purH*** | **0.349** | **0.00311** | **bifunctional phosphoribosylaminoimidazolecarboxamide formyltransferase/IMP cyclohydrolase** |
| **PA4855** | ***purD*** | **0.337** | **0.0021** | **phosphoribosylamine--glycine ligase** |
| PA4876 | *osmE* | 4.503 | 4.8E-05 | OsmE family transcriptional regulator |
| PA4880 | *-* | 6.887 | 7.7E-12 | bacterioferritin |
| **PA4891** | ***ureE*** | **0.446** | **0.04586** | **urease accessory protein UreE** |
| **PA4892** | ***ureF*** | **0.385** | **0.02198** | **urease accessory protein UreF** |
| **PA4893** | ***ureG*** | **0.416** | **0.02075** | **urease accessory protein UreG** |
| **PA4934** | ***rpsR*** | **0.368** | **0.04911** | **30S ribosomal protein S18** |
| **PA4935** | ***rpsF*** | **0.411** | **0.04714** | **30S ribosomal protein S6** |
| PA4942 | *hflK* | 2.386 | 0.04055 | protease subunit HflK |
| PA5025 | *metY* | 0.341 | 0.00119 | O-acetylhomoserine aminocarboxypropyltransferase |
| PA5046 | *-* | 0.254 | 0.00011 | malic enzyme |
| PA5078 | *-* | 2.32 | 0.04055 | glucan biosynthesis protein G |
| PA5128 | *secB* | 0.348 | 0.00697 | preprotein translocase subunit SecB |
| PA5157 | *-* | 0.33 | 0.00309 | transcriptional regulator |
| PA5159 | *-* | 0.428 | 0.02028 | multidrug resistance protein |
| PA5199 | *amgS* | 5.307 | 3.9E-09 | protein AmgS |
| PA5217 | *-* | 15.977 | 3.3E-25 | iron ABC transporter substrate-binding protein |
| PA5245 | *-* | 2.451 | 0.01237 | glutamine amidotransferase |
| PA5312 | *-* | 2.511 | 0.00511 | aldehyde dehydrogenase |
| PA5316 | *rpmB* | 0.304 | 0.00531 | 50S ribosomal protein L28 |
| **PA5365** | ***phoU*** | **2.762** | **0.0021** | **phosphate uptake regulatory protein PhoU** |
| **PA5366** | ***pstB*** | **3.425** | **6.5E-05** | **phosphate ABC transporter ATP-binding protein** |
| **PA5367** | ***pstA*** | **2.979** | **0.00107** | **phosphate ABC transporter permease** |
| **PA5368** | ***pstC*** | **3.104** | **0.00071** | **phosphate ABC transporter permease** |
| **PA5369** | ***pstS*** | **5.081** | **5.2E-08** | **phosphate ABC transporter substrate-binding protein** |
| PA5429 | *aspA* | 2.51 | 0.01251 | aspartate ammonia-lyase |
| PA5435 | *-* | 0.174 | 2.7E-07 | pyruvate carboxylase subunit B |
| PA5476 | *citA* | 0.431 | 0.02173 | citrate transporter |
| PA5483 | *algB* | 2.204 | 0.02787 | two-component response regulator AlgB |
| PA5489 | *dsbA* | 3.027 | 0.00442 | thiol:disulfide interchange protein DsbA |
| PA5521 | *-* | 2.281 | 0.04055 | short-chain dehydrogenase |
| PA5523 | *-* | 2.582 | 0.00788 | aminotransferase |
| PA5525 | *-* | 2.211 | 0.02581 | transcriptional regulator |
| PA5529 | *-* | 3.652 | 5.2E-05 | sodium/proton antiporter |
| PA5557 | *atpH* | 0.407 | 0.03446 | ATP synthase subunit delta |

^a^ Gene name, ORF number, and annotation are from the *Pseudomonas* genome project [48]. Differently transcribed operons are illustrated in bold.

^b^ Fold changes are mean values of three bioreplicates

Table S6 DEGs specifically regulated by *P. putida* KT2440^a^.

| ORF | Gene | Fold change^b^ | P_adjust_ | Annotation |
| --- | --- | --- | --- | --- |
| PP_0009 | *rpmH* | 2.89 | 0.037538 | 50S ribosomal protein L34 |
| PP_0024 | *-* | 15.04 | 5.68E-16 | lipid A phosphoethanolamine transferase |
| PP_0035 | *-* | 49.29 | 8.65E-43 | sugar translocase |
| PP_0036 | *-* | 101.42 | 8.28E-71 | LysR family transcriptional regulator |
| PP_0037 | *oprP* | 10.05 | 3.45E-31 | porin |
| PP_0041 | *cadA1* | 8.25 | 1.67E-51 | cadmium-transporting P-type ATPase CadA1 |
| PP_0046 | *opdT1* | 51.27 | 8.17E-164 | porin |
| PP_0047 | *czcR2* | 17.78 | 1.76E-37 | DNA-binding response regulator |
| **PP_0103** | ***-*** | **0.23** | **1.29E-33** | **cytochrome c oxidase subunit II** |
| **PP_0104** | ***ctaD*** | **0.22** | **6.81E-32** | **cytochrome c oxidase subunit I** |
| **PP_0105** | ***-*** | **0.17** | **1.81E-16** | **cytochrome c oxidase assembly protein** |
| **PP_0106** | ***-*** | **0.29** | **9.50E-20** | **MFS transporter** |
| **PP_0109** | ***-*** | **0.19** | **8.10E-30** | **cytochrome b561** |
| **PP_0110** | ***-*** | **0.25** | **2.31E-15** | **protoheme IX farnesyltransferase** |
| **PP_0117** | ***znuB*** | **0.39** | **0.000234** | **membrane protein** |
| **PP_0118** | ***znuC*** | **0.47** | **0.000503** | **zinc import ATP-binding protein ZnuC** |
| **PP_0120** | ***-*** | **0.40** | **2.44E-10** | **zinc ABC transporter substrate-binding protein** |
| PP_0130 | *amiD* | 0.36 | 2.87E-11 | N-acetylmuramoyl-L-alanine amidase |
| PP_0137 | *gltP* | 2.35 | 0.000395 | proton glutamate symport protein |
| PP_0154 | *scpC* | 6.57 | 8.16E-86 | acetyl-CoA hydrolase |
| **PP_0169** | ***-*** | **2.96** | **9.79E-05** | **taurine dioxygenase** |
| **PP_0170** | ***-*** | **2.94** | **0.000378** | **ABC transporter substrate-binding protein** |
| **PP_0172** | ***-*** | **2.35** | **0.004092** | **ABC transporter permease** |
| PP_0190 | *-* | 2.61 | 3.20E-06 | disulfide bond formation protein B 2 |
| **PP_0204** | ***-*** | **3.33** | **5.65E-05** | **GntR family transcriptional regulator** |
| **PP_0205** | ***-*** | **4.08** | **6.18E-13** | **oxidoreductase** |
| PP_0210 | *-* | 2.26 | 0.024704 | PBS lyase |
| PP_0213 | *gabD1* | 2.85 | 1.24E-46 | NAD-dependent succinate-semialdehyde dehydrogenase |
| PP_0214 | *gabT* | 3.42 | 6.15E-45 | 4-aminobutyrate aminotransferase |
| **PP_0220** | ***metNB*** | **5.91** | **7.29E-08** | **methionine import ATP-binding protein MetN 2** |
| **PP_0221** | ***-*** | **2.16** | **0.037064** | **methionine ABC transporter substrate-binding protein** |
| **PP_0222** | ***-*** | **4.11** | **1.22E-07** | **N5,N10-methylene tetrahydromethanopterin reductase** |
| **PP_0223** | ***-*** | **3.26** | **4.08E-05** | **SfnB family sulfur acquisition oxidoreductase** |
| **PP_0224** | ***-*** | **6.89** | **1.10E-22** | **SfnB family sulfur acquisition oxidoreductase** |
| PP_0227 | *fliY* | 0.43 | 2.53E-13 | cystine transporter subunit |
| **PP_0230** | ***atsK*** | **4.68** | **1.33E-11** | **taurine dioxygenase** |
| **PP_0231** | ***tauC*** | **3.48** | **8.06E-05** | **taurine ABC transporter permease** |
| **PP_0232** | ***tauB2*** | **4.20** | **5.03E-07** | **taurine import ATP-binding protein TauB** |
| **PP_0233** | ***tauA*** | **4.85** | **2.83E-16** | **taurine ABC transporter substrate-binding protein** |
| **PP_0237** | ***ssuA*** | **7.08** | **7.93E-28** | **ABC transporter substrate-binding protein** |
| **PP_0238** | ***ssuD*** | **9.52** | **4.05E-29** | **alkanesulfonate monooxygenase** |
| **PP_0239** | ***ssuC*** | **8.37** | **6.80E-16** | **sulfonate ABC transporter** |
| **PP_0240** | ***ssuB*** | **6.98** | **7.26E-21** | **aliphatic sulfonate ABC transporter ATP-binding protein** |
| PP_0241 | *-* | 4.56 | 0.00026 | transporter |
| **PP_0242** | ***-*** | **2.06** | **0.000473** | **TetR family transcriptional regulator** |
| **PP_0243** | ***gshA*** | **2.55** | **2.40E-20** | **glutamate--cysteine ligase** |
| PP_0247 | *envZ* | 2.51 | 2.82E-27 | two-component sensor histidine kinase |
| PP_0252 | *hslO* | 2.28 | 8.68E-18 | molecular chaperone Hsp33 |
| PP_0258 | *ygaU* | 0.49 | 1.22E-15 | peptidoglycan-binding protein LysM |
| PP_0269 | *-* | 31.61 | 2.45E-100 | glutamate synthase |
| PP_0270 | *-* | 2.04 | 0.018083 | two-component sensor histidine kinase |
| PP_0282 | *artJ* | 0.41 | 4.97E-18 | amino acid ABC transporter |
| **PP_0294** | ***cbcV*** | **0.43** | **2.02E-09** | **choline ABC transporter ATP-binding protein** |
| **PP_0295** | ***cbcW*** | **0.45** | **0.000363** | **choline ABC transporter permease subunit** |
| **PP_0296** | ***cbcX*** | **0.36** | **3.71E-14** | **glycine** |
| PP_0304 | *caiX* | 0.40 | 0.008471 | glycine |
| PP_0308 | *-* | 0.47 | 2.59E-06 | membrane dipeptidase |
| PP_0324 | *soxD* | 2.68 | 0.04201 | sarcosine oxidase subunit delta |
| PP_0328 | *fdhA* | 0.21 | 2.49E-36 | formaldehyde dehydrogenase, glutathione-independent |
| PP_0334 | *-* | 2.20 | 8.90E-08 | transposase |
| **PP_0353** | ***-*** | **23.77** | **2.78E-41** | **DNA polymerase III subunit epsilon** |
| **PP_0354** | ***-*** | **50.06** | **2.09E-186** | **CBS domain-containing protein** |
| **PP_0362** | ***bioB*** | **6.12** | **2.79E-30** | **biotin synthase BioB** |
| **PP_0363** | ***bioF*** | **4.60** | **6.72E-17** | **8-amino-7-oxononanoate synthase** |
| PP_0371 | *-* | 7.33 | 1.40E-11 | LysR family transcriptional regulator |
| PP_0372 | *aruC* | 71.16 | 9.65E-195 | acetylornithine aminotransferase |
| PP_0379 | *pqqB* | 2.24 | 6.26E-11 | pyrroloquinoline quinone biosynthesis protein B |
| **PP_0412** | ***-*** | **0.07** | **1.08E-216** | **spermidine** |
| **PP_0413** | ***-*** | **0.08** | **5.15E-93** | **polyamine ABC transporter substrate-binding protein** |
| **PP_0414** | ***-*** | **0.16** | **6.72E-67** | **polyamine ABC transporter permease** |
| PP_0487 | *-* | 2.18 | 0.048474 | membrane protein |
| PP_0495 | *ansA* | 2.31 | 0.000192 | L-asparaginase 1 |
| PP_0504 | *oprG* | 2.34 | 1.05E-17 | outer membrane protein W |
| PP_0506 | *-* | 0.42 | 0.00028 | peptide ABC transporter permease |
| PP_0544 | *-* | 0.48 | 0.005331 | ethanolamine permease |
| PP_0545 | *aldB1* | 0.18 | 9.07E-67 | aldehyde dehydrogenase |
| PP_0557 | *acoR* | 0.24 | 4.97E-29 | sigma-54-dependent Fis family transcriptional regulator |
| PP_0596 | *-* | 3.43 | 4.50E-27 | omega amino acid--pyruvate aminotransferase |
| PP_0597 | *mmsA1* | 2.26 | 3.80E-14 | methylmalonate-semialdehyde dehydrogenase (acylating) |
| PP_0620 | *-* | 9.03 | 2.99E-26 | GntR family transcriptional regulator |
| PP_0629 | *-* | 0.47 | 8.73E-06 | membrane protein |
| PP_0642 | *-* | 2.70 | 0.036819 | membrane protein |
| **PP_0656** | ***-*** | **2.06** | **0.004123** | **amino acid ABC transporter permease** |
| **PP_0657** | ***-*** | **2.83** | **2.46E-10** | **amino acid ABC transporter substrate-binding protein** |
| PP_0675 | *gdhA* | 2.76 | 5.33E-09 | glutamate dehydrogenase |
| PP_0676 | *-* | 2.12 | 0.000787 | transcription elongation factor GreAB |
| PP_0677 | *-* | 2.65 | 5.59E-11 | lipoprotein |
| PP_0699 | *-* | 7.84 | 8.72E-17 | lysine transporter LysE |
| **PP_0711** | ***ycaC1*** | **0.34** | **1.53E-21** | **hydrolase** |
| **PP_0712** | ***ppkB*** | **0.43** | **5.56E-20** | **polyphosphate kinase 2** |
| **PP_0713** | ***kefB1*** | **0.40** | **1.61E-14** | **potassium efflux system protein** |
| **PP_0714** | ***-*** | **0.35** | **2.02E-15** | **AI-2E family transporter** |
| **PP_0715** | ***-*** | **0.44** | **7.16E-12** | **RND transporter** |
| **PP_0716** | ***-*** | **0.37** | **5.93E-09** | **hemolysin D** |
| **PP_0717** | ***-*** | **0.47** | **0.000743** | **membrane protein** |
| PP_0763 | *-* | 0.38 | 1.20E-33 | long-chain-fatty-acid--CoA ligase |
| PP_0767 | *-* | 2.03 | 3.60E-21 | LuxR family transcriptional regulator |
| PP_0798 | *-* | 2.19 | 4.65E-10 | diguanylate cyclase |
| PP_0799 | *opdC* | 3.19 | 1.19E-12 | porin |
| **PP_0803** | ***-*** | **0.46** | **1.83E-19** | **HlyD family type I secretion periplasmic adaptor subunit** |
| **PP_0804** | ***-*** | **0.44** | **3.67E-27** | **ATP-binding protein** |
| **PP_0805** | ***-*** | **0.50** | **3.58E-15** | **channel protein TolC** |
| **PP_0806** | ***-*** | **0.37** | **8.75E-51** | **surface adhesion protein** |
| PP_0828 | *-* | 2.68 | 8.65E-08 | effector protein |
| PP_0843 | *iscU* | 2.27 | 1.94E-13 | iron-sulfur cluster scaffold-like protein |
| PP_0846 | *hscA* | 2.14 | 2.56E-23 | Fe-S protein assembly chaperone HscA |
| PP_0847 | *fdx* | 2.09 | 0.000694 | ferredoxin, 2Fe-2S type, ISC system |
| **PP_0880** | ***dppC*** | **0.48** | **0.000472** | **peptide transporter** |
| **PP_0881** | ***dppB*** | **0.47** | **0.000403** | **peptide ABC transporter permease** |
| **PP_0884** | ***dppA2*** | **0.43** | **0.002537** | **ABC transporter** |
| **PP_0885** | ***dppA3*** | **0.44** | **3.23E-10** | **ABC transporter** |
| PP_0897 | *-* | 2.07 | 2.99E-15 | fumarate hydratase |
| **PP_0906** | ***-*** | **0.35** | **2.63E-20** | **acriflavine resistance protein B** |
| **PP_0907** | ***-*** | **0.36** | **1.76E-11** | **MexH family multidrug efflux RND transporter periplasmic adaptor subunit** |
| PP_0944 | *fumC1* | 2.28 | 0.000161 | class II fumarate hydratase |
| PP_0986 | *gcvT1* | 0.50 | 6.61E-10 | glycine cleavage system protein T |
| **PP_0999** | ***arcC*** | **0.28** | **1.26E-50** | **carbamate kinase** |
| **PP_1000** | ***arcB*** | **0.26** | **8.21E-52** | **ornithine carbamoyltransferase** |
| **PP_1001** | ***arcA*** | **0.32** | **1.08E-13** | **arginine deiminase** |
| **PP_1002** | ***arcD1*** | **0.27** | **6.07E-63** | **arginine-ornithine antiporter** |
| **PP_1003** | ***arcD2*** | **0.33** | **1.89E-22** | **arginine-ornithine antiporter** |
| **PP_1015** | ***gtsA*** | **0.40** | **4.26E-46** | **sugar ABC transporter substrate-binding protein** |
| **PP_1016** | ***gtsB*** | **0.14** | **1.84E-43** | **sugar ABC transporter permease** |
| **PP_1017** | ***gtsC*** | **0.16** | **5.30E-26** | **sugar ABC transporter permease** |
| **PP_1018** | ***gtsD*** | **0.19** | **3.93E-54** | **sugar ABC transporter ATP-binding protein** |
| **PP_1019** | ***oprB1*** | **0.23** | **1.04E-44** | **porin** |
| **PP_1022** | ***zwfA*** | **2.09** | **1.63E-22** | **glucose-6-phosphate dehydrogenase** |
| **PP_1023** | ***pgl*** | **2.02** | **7.55E-19** | **6-phosphogluconolactonase** |
| **PP_1024** | ***eda*** | **2.35** | **9.16E-30** | **ketohydroxyglutarate aldolase** |
| PP_1033 | *-* | 0.45 | 1.29E-20 | sulfatase |
| PP_1057 | *-* | 2.10 | 0.001431 | PadR family transcriptional regulator |
| PP_1079 | *argF* | 2.14 | 5.14E-08 | ornithine carbamoyltransferase |
| PP_1082 | *Bfr2* | 2.11 | 3.15E-09 | bacterioferritin |
| PP_1094 | *-* | 0.49 | 0.007092 | electron transporter RnfG |
| PP_1116 | *-* | 2.96 | 0.00441 | serine recombinase |
| **PP_1121** | ***-*** | **0.24** | **1.39E-98** | **membrane protein** |
| **PP_1122** | ***-*** | **0.37** | **2.11E-25** | **membrane protein** |
| PP_1128 | *-* | 0.47 | 1.66E-18 | flagellar motor protein MotB |
| PP_1133 | *-* | 2.71 | 0.000819 | transposase |
| **PP_1137** | ***livF1*** | **0.43** | **0.000743** | **ABC transporter ATP-binding protein** |
| **PP_1139** | ***livM*** | **0.41** | **6.15E-08** | **branched-chain amino acid ABC transporter permease** |
| **PP_1140** | ***livH*** | **0.35** | **2.31E-05** | **branched-chain amino acid transporter permease subunit LivH** |
| **PP_1141** | ***livK*** | **0.31** | **3.25E-30** | **branched chain amino acid ABC transporter substrate-binding protein** |
| PP_1144 | *-* | 0.48 | 2.59E-20 | diguanylate cyclase |
| PP_1181 | *-* | 0.46 | 0.000694 | DNA-binding response regulator |
| PP_1192 | *-* | 0.49 | 7.76E-05 | acetyltransferase |
| PP_1210 | *dps* | 0.46 | 4.27E-17 | DNA starvation |
| PP_1254 | *xenA* | 5.69 | 1.45E-28 | NADH:flavin oxidoreductase |
| PP_1257 | *-* | 0.40 | 0.00241 | dihydrodipicolinate synthase family protein |
| PP_1260 | *-* | 2.71 | 0.000819 | transposase |
| **PP_1264** | ***-*** | **33.54** | **6.91E-138** | **fusaric acid resistance protein** |
| **PP_1266** | ***-*** | **32.66** | **8.15E-82** | **membrane protein** |
| PP_1267 | *yha* | 2.12 | 0.002791 | toxin endoribonuclease of toxin antitoxin system SohB(PrlF)-YhaV |
| **PP_1271** | ***-*** | **0.32** | **2.23E-14** | **EmrB** |
| **PP_1272** | ***-*** | **0.30** | **6.50E-19** | **DSBA oxidoreductase** |
| **PP_1273** | ***-*** | **0.30** | **1.76E-16** | **RND transporter** |
| PP_1297 | *yhdW* | 0.34 | 8.60E-08 | amino acid ABC transporter substrate-binding protein |
| **PP_1303** | ***cysD*** | **2.48** | **5.83E-29** | **sulfate adenylyltransferase subunit 2** |
| **PP_1304** | ***cysC*** | **2.11** | **2.24E-20** | **bifunctional sulfate adenylyltransferase subunit 1** |
| **PP_1318** | ***petB*** | **0.48** | **4.27E-17** | **cytochrome b** |
| **PP_1319** | ***petC*** | **0.47** | **4.91E-18** | **cytochrome c** |
| PP_1345 | *secA* | 2.50 | 1.90E-35 | preprotein translocase subunit SecA |
| PP_1376 | *pcaK* | 0.48 | 0.036908 | 4-hydroxybenzoate transporter |
| PP_1382 | *pcaP* | 0.49 | 0.00013 | membrane protein |
| PP_1403 | *bglX* | 2.59 | 2.72E-16 | beta-glucosidase |
| PP_1418 | *-* | 0.37 | 0.041239 | C4-dicarboxylate ABC transporter substrate-binding protein |
| PP_1440 | *cmoB* | 2.57 | 7.72E-07 | tRNA (mo5U34)-methyltransferase |
| PP_1444 | *gcd* | 0.46 | 7.58E-05 | glucose dehydrogenase |
| PP_1467 | *-* | 0.48 | 1.23E-11 | sodium:proton antiporter |
| **PP_1481** | ***patD*** | **0.33** | **5.93E-21** | **gamma-aminobutyraldehyde dehydrogenase** |
| **PP_1482** | ***ydcV*** | **0.37** | **2.91E-08** | **spermidine** |
| **PP_1483** | ***ydcU*** | **0.29** | **2.67E-08** | **spermidine** |
| **PP_1484** | ***ydcT*** | **0.27** | **1.83E-09** | **polyamine ABC transporter ATP-binding protein** |
| PP_1486 | *ydcS* | 0.28 | 8.52E-34 | spermidine |
| PP_1506 | *adk* | 3.56 | 8.89E-31 | adenylate kinase |
| PP_1559 | *-* | 0.32 | 0.015498 | holin |
| PP_1587 | *-* | 2.14 | 9.50E-11 | Na+ |
| PP_1635 | *-* | 2.09 | 3.21E-09 | DNA-binding response regulator |
| PP_1637 | *-* | 3.41 | 4.18E-27 | LysR family transcriptional regulator |
| PP_1638 | *Fpr1* | 4.79 | 7.97E-52 | ferredoxin--NADP(+) reductase |
| PP_1661 | *-* | 0.37 | 5.53E-45 | dehydrogenase |
| PP_1670 | *-* | 0.28 | 2.63E-20 | peptidase P60 |
| PP_1682 | *-* | 2.99 | 1.06E-15 | glycosyl hydrolase |
| PP_1689 | *-* | 0.37 | 1.82E-24 | aromatic hydrocarbon degradation protein |
| PP_1726 | *-* | 0.35 | 1.81E-32 | ABC transporter substrate-binding protein |
| PP_1741 | *betX* | 0.49 | 3.18E-17 | glycine |
| PP_1811 | *rffE* | 0.42 | 7.95E-13 | UDP-N-acetyl glucosamine 2-epimerase |
| PP_1816 | *-* | 0.47 | 2.23E-13 | NADP-dependent oxidoreductase |
| PP_1829 | *-* | 2.28 | 1.22E-07 | alpha |
| PP_1852 | *-* | 0.46 | 1.47E-07 | 3-ketoacyl-ACP reductase |
| **PP_1929** | ***arsB1*** | **2.90** | **0.000218** | **arsenic transporter** |
| **PP_1930** | ***arsR1*** | **4.96** | **2.82E-07** | **transcriptional regulator** |
| PP_1979 | *-* | 2.19 | 0.00038 | hydrolase |
| PP_1986 | *leuD* | 2.26 | 4.29E-11 | 3-isopropylmalate dehydratase small subunit |
| PP_2007 | *-* | 0.26 | 3.71E-36 | P-47 |
| PP_2018 | *-* | 0.38 | 8.65E-05 | BNR |
| PP_2023 | *-* | 2.44 | 0.000756 | glutathione S-transferase |
| **PP_2036** | ***-*** | **3.32** | **2.06E-13** | **dihydrodipicolinate synthase family protein** |
| **PP_2037** | ***--*** | **6.18** | **6.49E-14** | **class II aldolase** |
| **PP_2047** | ***-*** | **0.43** | **0.000827** | **3-hydroxyacyl-CoA dehydrogenase** |
| **PP_2048** | ***-*** | **0.47** | **0.00229** | **acyl-CoA dehydrogenase** |
| **PP_2049** | ***-*** | **0.45** | **0.006265** | **alcohol dehydrogenase** |
| **PP_2051** | ***fadA*** | **0.47** | **0.000335** | **acetyl-CoA acetyltransferase** |
| PP_2156 | *lolE* | 2.39 | 1.65E-14 | multidrug ABC transporter substrate-binding protein |
| **PP_2183** | ***-*** | **0.37** | **1.91E-05** | **formate dehydrogenase subunit gamma** |
| **PP_2184** | ***-*** | **0.42** | **4.91E-15** | **formate dehydrogenase subunit beta** |
| **PP_2185** | ***-*** | **0.48** | **1.63E-15** | **formate dehydrogenase subunit alpha** |
| PP_2201 | *-* | 3.05 | 6.23E-17 | alpha |
| PP_2206 | *yegQ* | 2.79 | 9.46E-06 | U32 family peptidase |
| PP_2233 | *-* | 0.43 | 9.83E-21 | isochorismatase |
| PP_2244 | *-* | 2.05 | 7.20E-14 | membrane protein |
| **PP_2246** | ***dauA*** | **0.39** | **6.59E-06** | **FAD-dependent oxidoreductase** |
| **PP_2247** | ***-*** | **0.37** | **0.001344** | **enamine deaminase RidA** |
| PP_2258 | *-* | 2.19 | 2.38E-05 | diguanylate cyclase |
| PP_2259 | *-* | 0.35 | 7.42E-14 | sigma-54-dependent Fis family transcriptional regulator |
| PP_2264 | *-* | 0.50 | 0.000311 | ABC transporter substrate-binding protein |
| PP_2299 | *tig* | 2.01 | 5.75E-19 | trigger factor |
| PP_2310 | *-* | 2.00 | 2.72E-10 | chemotaxis protein |
| PP_2358 | *-* | 0.35 | 4.92E-28 | putative Type 1 pili subunit |
| PP_2359 | *-* | 0.35 | 6.67E-24 | putative Type 1 pili subunit |
| **PP_2360** | ***-*** | **0.37** | **6.80E-18** | **putative Type 1 pili subunit** |
| **PP_2361** | ***-*** | **0.36** | **8.10E-30** | **type 1 pili usher pathway chaperone CsuC** |
| **PP_2362** | ***-*** | **0.37** | **1.60E-29** | **fimbrial biogenesis outer membrane usher protein** |
| PP_2377 | *-* | 2.29 | 3.11E-05 | acyltransferase |
| PP_2378 | *nfuA* | 2.66 | 3.37E-15 | Fe-S biogenesis protein NfuA |
| PP_2379 | *-* | 9.82 | 4.40E-58 | photosynthetic protein synthase I |
| PP_2408 | *czcC2* | 2.47 | 0.008826 | cobalt-zinc-cadmium resistance protein CzcC |
| PP_2432 | *nfnB* | 2.42 | 3.61E-07 | oxygen-insensitive NAD(P)H-dependent nitroreductase NfsB |
| **PP_2439** | ***ahpC*** | **5.51** | **1.46E-24** | **peroxiredoxin** |
| **PP_2440** | ***ahpF*** | **4.46** | **2.29E-14** | **alkyl hydroperoxide reductase subunit F** |
| **PP_2454** | ***rbsB*** | **0.21** | **4.13E-07** | **monosaccharide-transporting ATPase** |
| **PP_2455** | ***rbsA1*** | **0.35** | **0.003334** | **sugar ABC transporter ATPase** |
| PP_2476 | *curA* | 3.27 | 2.76E-05 | NADP-dependent oxidoreductase |
| PP_2492 | *yqhD* | 2.09 | 0.000942 | alcohol dehydrogenase |
| PP_2559 | *hasE* | 0.49 | 0.016462 | HlyD family type I secretion periplasmic adaptor subunit |
| PP_2569 | *-* | 0.40 | 1.01E-15 | MFS transporter |
| PP_2577 | *-* | 0.43 | 9.93E-19 | mammalian cell entry protein |
| PP_2584 | *oguA* | 2.25 | 2.01E-07 | hydroxydechloroatrazine ethylaminohydrolase |
| **PP_2656** | ***pstS*** | **2.81** | **1.98E-20** | **phosphate ABC transporter substrate-binding protein** |
| **PP_2657** | ***pstC*** | **2.44** | **5.11E-10** | **phosphate ABC transporter permease subunit PstC** |
| **PP_2658** | ***pstA*** | **2.90** | **1.22E-10** | **phosphate ABC transporter, permease protein PstA** |
| **PP_2659** | ***pstB1*** | **2.58** | **1.22E-09** | **phosphate ABC transporter ATP-binding protein** |
| PP_2730 | *-* | 2.23 | 4.44E-08 | lipoprotein |
| PP_2734 | *cfa* | 2.07 | 0.000167 | cyclopropane-fatty-acyl-phospholipid synthase |
| **PP_2737** | ***-*** | **2.46** | **8.54E-10** | **short-chain dehydrogenase** |
| **PP_2738** | ***-*** | **2.32** | **1.62E-06** | **transcriptional regulator** |
| PP_2790 | *-* | 2.90 | 2.19E-11 | propionate catabolism operon regulatory protein PrpR |
| PP_2861 | *-* | 3.84 | 6.37E-15 | methyl-accepting chemotaxis sensory transducer |
| PP_2869 | *-* | 6.35 | 5.67E-16 | oxidoreductase |
| **PP_2870** | ***-*** | **8.18** | **9.37E-17** | **spermidine** |
| **PP_2871** | ***-*** | **2.35** | **0.028085** | **class II aldolase** |
| PP_2876 | *ampC* | 2.40 | 3.29E-09 | class C beta-lactamase |
| PP_2880 | *-* | 2.45 | 0.00229 | TetR family transcriptional regulator |
| PP_2913 | *hemB* | 2.13 | 5.80E-20 | delta-aminolevulinic acid dehydratase |
| PP_2918 | *treSA* | 2.05 | 5.49E-08 | trehalose synthase |
| PP_2936 | *ybiT* | 2.08 | 0.00016 | ABC-F family ATPase |
| PP_2940 | *yefM* | 2.19 | 0.006976 | prevent-host-death protein |
| PP_2951 | *-* | 2.12 | 0.031343 | TetR family transcriptional regulator |
| PP_2988 | *-* | 4.07 | 0.00016 | NADPH:quinone oxidoreductase |
| PP_3016 | *-* | 2.19 | 2.70E-05 | LPS biosynthesis protein |
| PP_3093 | *-* | 2.01 | 2.59E-08 | type VI secretion protein |
| **PP_3098** | ***-*** | **2.85** | **2.61E-06** | **type VI secretion protein** |
| **PP_3099** | ***puuD*** | **2.26** | **2.66E-19** | **EvpB family type VI secretion protein** |
| **PP_3100** | ***-*** | **2.51** | **4.24E-14** | **type VI secretion protein** |
| PP_3122 | *atoA* | 3.67 | 7.05E-08 | 3-oxoacid CoA-transferase subunit A |
| PP_3127 | *-* | 0.49 | 1.23E-15 | LPS biosynthesis protein |
| PP_3152 | *yneJ* | 2.17 | 0.005264 | LysR family transcriptional regulator |
| PP_3170 | *-* | 2.29 | 0.000405 | LysR family transcriptional regulator |
| PP_3225 | *-* | 2.35 | 0.001012 | dihydrofolate reductase |
| PP_3229 | *-* | 2.50 | 0.043384 | ABC transporter substrate-binding protein |
| PP_3267 | *-* | 2.01 | 0.027506 | Clp protease |
| PP_3287 | *fnrC* | 2.42 | 3.03E-05 | Crp |
| PP_3305 | *-* | 3.21 | 0.000475 | membrane protein |
| PP_3332 | *-* | 5.99 | 3.62E-07 | cytochrome |
| **PP_3342** | ***nikA*** | **3.78** | **5.49E-08** | **nickel ABC transporter, nickel** |
| **PP_3343** | ***nikB*** | **2.95** | **0.007462** | **nickel ABC transporter permease subunit NikB** |
| **PP_3344** | ***nikC*** | **2.64** | **0.003718** | **nickel ABC transporter permease subunit NikC** |
| **PP_3345** | ***nikD*** | **2.82** | **0.001482** | **nickel import ATP-binding protein NikD** |
| **PP_3346** | ***nikE*** | **4.05** | **5.92E-06** | **nickel import ATP-binding protein NikE** |
| **PP_3382** | ***-*** | **2.49** | **3.86E-15** | **gluconate 2-dehydrogenase** |
| **PP_3383** | ***-*** | **2.35** | **8.59E-13** | **GMC family oxidoreductase** |
| **PP_3384** | ***-*** | **2.04** | **6.74E-05** | **gluconate 2-dehydrogenase** |
| PP_3394 | *-* | 2.38 | 0.003091 | hydroxymethylglutaryl-CoA lyase |
| PP_3405 | *-* | 2.77 | 8.00E-06 | lysine transporter LysE |
| PP_3433 | *hpd* | 0.47 | 1.92E-05 | 4-hydroxyphenylpyruvate dioxygenase |
| PP_3436 | *rarD* | 2.77 | 5.67E-18 | permease |
| PP_3446 | *ilvA1* | 2.66 | 3.25E-06 | L-threonine dehydratase biosynthetic IlvA |
| PP_3458 | *acs* | 0.31 | 3.06E-10 | AMP-binding protein |
| PP_3503 | *-* | 0.28 | 2.88E-16 | sigma-54-dependent Fis family transcriptional regulator |
| PP_3516 | *-* | 0.41 | 0.00042 | AraC family transcriptional regulator |
| **PP_3528** | ***-*** | **4.88** | **3.22E-10** | **ABC transporter substrate-binding protein** |
| **PP_3529** | ***dmoA2*** | **2.57** | **4.44E-05** | **N5,N10-methylene tetrahydromethanopterin reductase** |
| PP_3564 | *-* | 2.09 | 8.22E-05 | AraC family transcriptional regulator |
| PP_3593 | *-* | 2.51 | 0.002376 | amino acid ABC transporter |
| PP_3596 | *amaD* | 2.52 | 0.014707 | D-amino-acid dehydrogenase |
| PP_3622 | *-* | 0.47 | 1.29E-19 | isoquinoline 1-oxidoreductase subunit beta |
| **PP_3623** | ***adhB*** | **0.48** | **5.02E-12** | **gluconate 2-dehydrogenase** |
| **PP_3633** | ***-*** | **2.20** | **0.003329** | **N-acetyl-gamma-glutamyl-phosphate reductase 2** |
| PP_3639 | *-* | 4.43 | 5.57E-05 | alkylhydroperoxidase |
| PP_3693 | *-* | 0.50 | 6.01E-05 | transcriptional regulator |
| PP_3764 | *opdN* | 5.15 | 5.99E-14 | porin |
| PP_3814 | *-* | 0.38 | 0.000135 | ABC transporter substrate-binding protein |
| PP_3817 | *-* | 0.45 | 0.001482 | ABC transporter |
| PP_3823 | *-* | 2.01 | 0.001421 | cytochrome c |
| PP_3899 | *-* | 2.52 | 0.002537 | HicB |
| PP_3922 | *sohB* | 2.08 | 1.74E-16 | protease |
| **PP_3954** | ***-*** | **0.24** | **1.18E-19** | **C4-dicarboxylate ABC transporter** |
| **PP_3955** | ***-*** | **0.30** | **5.44E-11** | **C4-dicarboxylate ABC transporter** |
| PP_3958 | *nhaA2* | 2.72 | 5.93E-21 | Na+ |
| PP_4010 | *cspD* | 0.33 | 5.57E-16 | cold shock domain protein CspD |
| PP_4021 | *cpo* | 0.48 | 6.07E-10 | alpha |
| **PP_4033** | ***rnz*** | **0.19** | **4.44E-64** | **ribonuclease Z** |
| **PP_4034** | ***hyuC*** | **0.14** | **4.00E-156** | **Zn-dependent hydrolase** |
| **PP_4035** | ***pydP*** | **0.16** | **5.69E-121** | **nitrate reductase** |
| **PP_4036** | ***pydB*** | **0.24** | **4.87E-92** | **phenylhydantoinase** |
| **PP_4037** | ***pydX*** | **0.16** | **1.13E-120** | **oxidoreductase** |
| **PP_4038** | ***pydA*** | **0.17** | **1.06E-132** | **dihydropyrimidine dehydrogenase subunit B** |
| PP_4057 | *-* | 0.45 | 0.005523 | membrane protein |
| PP_4065 | *mccB* | 0.36 | 1.44E-05 | methylcrotonoyl-CoA carboxylase |
| PP_4068 | *-* | 2.31 | 2.96E-06 | Cro |
| PP_4111 | *fusB* | 0.44 | 9.13E-27 | elongation factor G |
| PP_4116 | *aceA* | 3.27 | 8.01E-17 | isocitrate lyase |
| PP_4247 | *-* | 0.46 | 7.84E-10 | exonuclease |
| **PP_4250** | ***ccoN2*** | **3.50** | **9.00E-29** | **cytochrome c oxidase, cbb3-type subunit I** |
| **PP_4251** | ***ccoO2*** | **5.36** | **9.41E-17** | **peptidase S41** |
| **PP_4253** | ***ccoP2*** | **4.98** | **6.26E-30** | **cytochrome c oxidase, cbb3-type subunit III** |
| **PP_4255** | ***ccoN2*** | **0.42** | **1.27E-23** | **cytochrome c oxidase, cbb3-type subunit I** |
| **PP_4256** | ***-*** | **0.47** | **3.00E-12** | **peptidase S41** |
| **PP_4257** | ***ccoQ-II*** | **0.31** | **0.003379** | **cytochrome oxidase** |
| **PP_4258** | ***ccoP2*** | **0.45** | **4.07E-19** | **cytochrome c oxidase, cbb3-type subunit III** |
| PP_4282 | *aqpZ* | 0.24 | 2.94E-40 | aquaporin |
| PP_4284 | *-* | 0.47 | 0.003934 | guanine permease |
| PP_4297 | *gcl* | 4.81 | 1.57E-18 | glyoxylate carboligase |
| PP_4315 | *-* | 2.44 | 2.63E-10 | isomerase |
| PP_4364 | *-* | 0.48 | 3.30E-07 | anti-sigma-factor antagonist |
| **PP_4401** | ***bkdAA*** | **0.41** | **0.000439** | **2-oxoisovalerate dehydrogenase** |
| **PP_4402** | ***bkdAB*** | **0.48** | **0.006408** | **2-oxoisovalerate dehydrogenase subunit beta** |
| **PP_4404** | ***lpdV*** | **0.44** | **1.18E-07** | **dihydrolipoyl dehydrogenase** |
| PP_4425 | *-* | 0.38 | 0.033923 | ectoine |
| PP_4519 | *tolC* | 0.47 | 5.11E-18 | channel protein TolC |
| PP_4540 | *-* | 0.50 | 1.17E-06 | 3-oxoadipate enol-lactonase |
| **PP_4575** | ***-*** | **0.46** | **2.26E-09** | **allophanate hydrolase** |
| **PP_4576** | ***ybgJ*** | **0.48** | **2.16E-15** | **allophanate hydrolase** |
| **PP_4578** | ***-*** | **0.32** | **2.12E-07** | **MFS transporter** |
| PP_4603 | *-* | 2.71 | 0.000819 | transposase |
| PP_4615 | *-* | 2.15 | 0.000219 | phosphate starvation-inducible protein PsiE |
| PP_4619 | *hmgC* | 0.47 | 0.012853 | maleylacetoacetate isomerase |
| PP_4636 | *yqeF* | 3.04 | 5.40E-08 | acetyl-CoA acetyltransferase |
| PP_4641 | *yjiY* | 0.35 | 1.34E-45 | carbon starvation protein A |
| PP_4655 | *pcaG* | 0.36 | 0.016525 | protocatechuate 3,4-dioxygenase subunit alpha |
| PP_4657 | *ypfJ* | 0.46 | 6.82E-21 | metallopeptidase |
| PP_4659 | *ggt* | 0.38 | 1.37E-13 | Gamma-glutamyltranspeptidase |
| PP_4678 | *ilvC* | 2.24 | 2.51E-21 | ketol-acid reductoisomerase |
| PP_4702 | *acsA2* | 0.48 | 2.03E-15 | acetyl-coenzyme A synthetase |
| PP_4756 | *gabP* | 0.49 | 0.003371 | GABA permease |
| PP_4758 | *-* | 0.45 | 7.27E-06 | MFS transporter |
| PP_4791 | *-* | 2.71 | 0.000819 | transposase |
| PP_4817 | *-* | 0.44 | 2.01E-10 | MaoC family dehydratase |
| PP_4851 | *-* | 0.38 | 2.75E-17 | phosphate starvation-inducible protein PsiF |
| **PP_4864** | ***braF*** | **0.45** | **0.001735** | **ABC transporter ATP-binding protein** |
| **PP_4865** | ***braE*** | **0.38** | **2.50E-10** | **branched-chain amino acid ABC transporter permease** |
| PP_4867 | *-* | 0.34 | 4.29E-11 | branched chain amino acid ABC transporter substrate-binding protein |
| **PP_4881** | ***-*** | **0.42** | **1.17E-26** | **sugar ABC transporter substrate-binding protein** |
| **PP_4882** | ***-*** | **0.39** | **9.09E-30** | **iron ABC transporter permease** |
| PP_4938 | *-* | 0.49 | 3.90E-13 | glycosyl transferase |
| PP_4946 | *putP* | 0.29 | 5.07E-10 | sodium:proline symporter |
| PP_4948 | *-* | 0.47 | 2.65E-05 | acyl-CoA dehydrogenase |
| PP_4983 | *-* | 0.48 | 6.92E-10 | amine oxidase |
| PP_5033 | *hutU* | 2.07 | 0.009104 | urocanate hydratase |
| PP_5037 | *-* | 2.14 | 0.000209 | lipocalin |
| PP_5046 | *glnA* | 2.73 | 2.57E-26 | type I glutamate--ammonia ligase |
| **PP_5075** | ***gltD*** | **2.10** | **7.48E-11** | **glutamate synthase** |
| **PP_5076** | ***gltB*** | **2.07** | **7.31E-20** | **glutamate synthase** |
| PP_5094 | *-* | 2.17 | 9.38E-09 | YggS family pyridoxal phosphate enzyme |
| **PP_5120** | ***calB*** | **0.38** | **1.79E-15** | **coniferyl-aldehyde dehydrogenase** |
| **PP_5122** | ***-*** | **0.32** | **3.67E-14** | **GMC family oxidoreductase** |
| PP_5130 | *pchP* | 0.45 | 8.82E-05 | phosphorylcholine phosphatase |
| PP_5165 | *plpB* | 5.00 | 5.31E-21 | methionine ABC transporter substrate-binding protein |
| PP_5166 | *-* | 4.54 | 3.58E-06 | Fis family transcriptional regulator |
| **PP_5168** | ***cysA*** | **2.21** | **3.10E-10** | **sulfate** |
| **PP_5170** | ***cysU*** | **3.17** | **1.47E-05** | **sulfate ABC transporter permease subunit CysT** |
| **PP_5171** | ***Sbp2*** | **4.98** | **1.23E-48** | **sulfate ABC transporter substrate-binding protein** |
| **PP_5177** | ***spuH*** | **0.44** | **1.74E-10** | **ornithine carbamoyltransferase** |
| **PP_5178** | ***spuG*** | **0.38** | **2.01E-10** | **spermidine** |
| **PP_5179** | ***spuF*** | **0.44** | **5.93E-13** | **putrescine** |
| **PP_5180** | ***spuE*** | **0.39** | **4.21E-11** | **ABC transporter substrate-binding protein** |
| **PP_5181** | ***spuD*** | **0.37** | **8.52E-37** | **spermidine** |
| PP_5210 | *-* | 0.48 | 1.31E-10 | NADPH:quinone oxidoreductase |
| PP_5241 | *-* | 0.44 | 2.83E-08 | LuxR family transcriptional regulator |
| PP_5256 | *-* | 0.50 | 0.001706 | mechanosensitive ion channel protein MscS |
| PP_5268 | *puuR* | 3.38 | 2.01E-06 | transcriptional regulator, XRE family |
| **PP_5269** | ***dadX*** | **2.24** | **0.00017** | **alanine racemase** |
| **PP_5270** | ***dadA2*** | **3.04** | **5.78E-18** | **D-amino acid dehydrogenase small subunit** |
| PP_5313 | *hupA* | 0.42 | 0.000317 | integration host factor |
| PP_5323 | *-* | 0.43 | 8.23E-16 | peptidase M23 |
| PP_5339 | *oruR* | 0.38 | 0.009138 | AraC family transcriptional regulator |

^a^ Gene name, ORF number, and annotation are from the *Pseudomonas* genome project [48]. Differently transcribed operons are illustrated in bold.

^b^ Fold changes are mean values of three bioreplicates

Table S7 DEGs specifically regulated by *P. fluorescence* ATCC13525^a^.

| ORF | Gene | Fold change^b^ | P_adjust_ | Gene description |
| --- | --- | --- | --- | --- |
| RS02275 | - | 0.482 | 0.003081992 | NarK/NasA family nitrate transporter |
| **RS02875** | **-** | **2.512** | **1.51049E-41** | **HAMP domain-containing protein** |
| **RS02880** | **-** | **2.376** | **9.01132E-45** | **DNA-binding response regulator** |
| RS05810 | - | 2.162 | 9.52485E-22 | phospholipid carrier-dependent glycosyltransferase |
| RS07670 |  | 2.054 | 2.00985E-13 | FAD-dependent oxireductase |
| RS08555 | - | 2.087 | 2.38586E-17 | nucleotide sugar aminotransferase |
| RS08565 | - | 2.068 | 3.55824E-28 | GNAT family N-acetyltransferase |
| RS11290 | - | 0.496 | 0.004447532 | energy transducer TonB |
| RS15310 | - | 2.072 | 4.39835E-24 | acyl-CoA dehydrogenase |
| RS16140 | - | 0.486 | 0.001938023 | outer membrane usher protein |
| **RS16750** | **-** | **2.084** | **7.63013E-10** | **amino acid ABC transporter ATP-binding protein** |
| **RS16755** | **-** | **2.254** | **7.24534E-06** | **amino acid ABC transporter permease** |
| **RS16760** | **-** | **2.391** | **9.92798E-08** | **amino acid ABC transporter permease** |
| **RS16765** | **-** | **2.112** | **2.7692E-19** | **amino acid ABC transporter substrate-binding protein** |
| **RS16770** | **-** | **2.001** | **2.00943E-30** | **glycerol-3-phosphate dehydrogenase** |
| **RS16780** | **glpK** | **3.334** | **2.04716E-60** | **glycerol kinase** |
| **RS16785** | **-** | **2.246** | **1.97885E-29** | **aquaporin** |
| RS18860 | - | 2.088 | 2.43094E-09 | class I SAM-dependent methyltransferase |
| **RS19050** | **fadB** | **2.702** | **5.00212E-64** | **multifunctional fatty acid oxidation complex subunit** |
| **RS19055** | **fadA** | **2.592** | **1.88871E-48** | **acetyl-CoA C-acyltransferase FadA** |
| RS19090 | - | 4.46 | 4.62523E-71 | TetR/AcrR family transcriptional regulator |
| RS20205 | - | 2.497 | 9.1841E-22 | glycine/betaine ABC transporter substrate-binding protein |
| RS21750 | - | 2.124 | 7.27028E-31 | iron ABC transporter substrate-binding protein |
| RS22445 | - | 2.832 | 1.06612E-29 | histidine phosphatase family protein |
| RS23460 | - | 2.053 | 1.72101E-15 | alkane 1-monooxygenase |
| RS24060 | - | 0.49 | 0.003506551 | ABC transporter substrate-binding protein |
| **RS24955** | **hcp** | **0.2** | **2.90507E-32** | **type VI secretion system tube protein Hcp** |
| **RS24975** | **-** | **0.365** | **4.45957E-06** | **DotU family type IV/VI secretion system protein** |
| **RS24980** | **tssK** | **0.257** | **6.81013E-18** | **type VI secretion system baseplate subunit TssK** |
| **RS24985** | **tssJ** | **0.328** | **8.03006E-08** | **type VI secretion system lipoprotein TssJ** |
| **RS24990** | **-** | **0.471** | **0.0021905** | **DUF4150 domain-containing protein** |
| **RS24995** | **-** | **0.302** | **6.94122E-09** | **DUF3540 domain-containing protein** |
| **RS25005** | **-** | **0.166** | **8.27079E-34** | **DUF2169 domain-containing protein** |
| **RS25010** | **vgrG** | **0.175** | **1.16837E-35** | **type VI secretion system tip protein VgrG** |
| **RS25015** | **tssG** | **0.421** | **0.000115836** | **type VI secretion system baseplate subunit TssG** |
| **RS25020** | **vasA** | **0.346** | **5.82122E-09** | **type VI secretion system baseplate subunit TssF** |
| **RS25030** | **tssC** | **0.15** | **7.71363E-44** | **type VI secretion system contractile sheath large subunit** |
| **RS25035** | **tssB** | **0.255** | **4.28668E-11** | **type VI secretion system contractile sheath small subunit** |
| **RS25040** | **tssA** | **0.197** | **1.5019E-22** | **type VI secretion system protein TssA** |
| RS27775 | - | 2.018 | 5.83796E-11 | glucose/quinate/shikimate family membrane-bound PQQ-dependent dehydrogenase |
| RS29015 | emrB | 2.08 | 4.78629E-22 | MFS transporter |
| RS29030 | - | 3.403 | 4.12361E-41 | MarR family transcriptional regulator |
| RS29665 | - | 3.061 | 4.85696E-24 | histidine phosphatase family protein |
| RS29675 | - | 2.283 | 1.64278E-20 | phospholipid carrier-dependent glycosyltransferase |

^a^ Gene name, ORF number, and annotation are from the *Pseudomonas* genome project [48]. Differently transcribed operons are illustrated in bold.

^b^ Fold changes are mean values of three bioreplicates

Table S8 Summary of significantly changed metabolites identified in *P. aeruginosa* PAO1

| Adduct | Description | Fold change^a^ | P_adj_ | SubClass |
| --- | --- | --- | --- | --- |
| M+ | Trigonelline | 0.10 | 7.93E-03 |  |
| (M+H)+ | all cis-(6,9,12)-Linolenic acid | 0.35 | 1.09E-02 |  |
| (M+H)+ | Pyridoxine | 2.65 | 2.57E-04 |  |
| (M+H-H_2_O)+ | Adenosine | 4.10 | 3.12E-02 |  |
| (M+H-H_2_O)+ | Deoxygalactonojirimycin | 4.66 | 2.98E-06 |  |
| (M-H)- | D-Glucono-1,5-lactone | 2.08 | 9.61E-02 |  |
| (M+H)+ | S-Methyl-5'-thioadenosine | 0.47 | 7.27E-03 | 5'-deoxy-5'-thionucleosides |
| (M-H)- | Pantothenate | 0.42 | 9.12E-03 | Alcohols and polyols |
| (M-H)- | DL-lactate | 0.40 | 7.94E-02 | Alpha hydroxy acids and derivatives |
| (M+CH_3_COO+2H)+ | L-Pyroglutamic acid | 0.17 | 5.36E-04 | Amino acids, peptides, and analogues |
| (M+H-H_2_O)+ | N-Acetyl-L-glutamate | 0.18 | 4.27E-04 | Amino acids, peptides, and analogues |
| (2M+Na)+ | L-Lysine | 0.48 | 5.44E-04 | Amino acids, peptides, and analogues |
| (M+H)+ | D-Proline | 2.02 | 3.14E-03 | Amino acids, peptides, and analogues |
| (M+H-H_2_O)+ | L-Glutamine | 2.60 | 1.73E-04 | Amino acids, peptides, and analogues |
| (M+H)+ | 3-Aminobutanoic acid | 6.86 | 2.95E-02 | Amino acids, peptides, and analogues |
| (M+H)+ | L-Proline | 11.01 | 3.72E-05 | Amino acids, peptides, and analogues |
| (M-H)- | N-Acetyl-L-glutamate | 0.14 | 3.93E-05 | Amino acids, peptides, and analogues |
| (M-H)- | L-Glutamate | 0.31 | 1.81E-03 | Amino acids, peptides, and analogues |
| (M+H-2H_2_O)+ | N-Acetyl-D-glucosamine | 0.10 | 7.32E-03 | Carbohydrates and carbohydrate conjugates |
| (M-H)- | DL-2-Phosphoglycerate | 0.08 | 2.37E-03 | Carbohydrates and carbohydrate conjugates |
| (M-H)- | N-Acetylglucosamine 1-phosphate | 0.13 | 4.17E-03 | Carbohydrates and carbohydrate conjugates |
| (M-H_2_O-H)- | Ribitol | 0.36 | 1.96E-04 | Carbohydrates and carbohydrate conjugates |
| (M-H_2_O-H)- | L-Sorbose | 0.37 | 6.78E-02 | Carbohydrates and carbohydrate conjugates |
| (M-H)- | Dihydroxyacetone phosphate | 0.45 | 3.27E-02 | Carbohydrates and carbohydrate conjugates |
| (M-H)- | 2-Methyl-3-hydroxybutyric acid | 0.37 | 2.51E-03 | Fatty acids and conjugates |
| (M-H)- | Hydroxyisocaproic acid | 0.39 | 3.62E-03 | Fatty acids and conjugates |
| (M+NH_4_)+ | L-Gulonic gamma-lactone | 0.42 | 4.51E-02 | Gamma butyrolactones |
| (M+NH_4_-2H)- | L-Gulonic gamma-lactone | 0.38 | 5.18E-02 | Gamma butyrolactones |
| (M+H)+ | Glycerol 3-phosphate | 0.29 | 8.55E-03 | Glycerophosphates |
| (M+H-H_2_O)+ | 5-Hydroxyindoleacetate | 0.49 | 5.37E-02 | Indolyl carboxylic acids and derivatives |
| (M+H-2H_2_O)+ | 3-Hydroxycapric acid | 0.45 | 7.60E-03 | Medium-chain hydroxy acids and derivatives |
| (M-H)- | 3-Hydroxycapric acid | 0.29 | 1.87E-02 | Medium-chain hydroxy acids and derivatives |
| (M-H)- | 3-Hydroxydodecanoic acid | 0.38 | 5.03E-03 | Medium-chain hydroxy acids and derivatives |
| (M+H-H_2_O)+ | Tyramine | 2.56 | 3.99E-04 | Phenethylamines |
| (M-H)- | Phosphoenolpyruvate | 0.16 | 6.52E-03 | Phosphate esters |
| (M+H)+ | Adenosine monophosphate | 0.47 | 2.56E-03 | Purine ribonucleotides |
| (M-H)- | Adenosine monophosphate | 0.41 | 6.12E-03 | Purine ribonucleotides |
| (M+H)+ | Hypoxanthine | 0.28 | 5.05E-03 | Purines and purine derivatives |
| (M-H)- | Hypoxanthine | 0.34 | 6.11E-03 | Purines and purine derivatives |
| (M+H)+ | Nicotinamide | 0.49 | 1.76E-02 | Pyridinecarboxylic acids and derivatives |
| (2M+H)+ | Deoxycytidine | 10.08 | 4.81E-03 | Pyrimidine 2'-deoxyribonucleosides |
| (M-H)- | Uridine 5'-monophosphate | 0.47 | 1.70E-02 | Pyrimidine ribonucleotides |
| (M+CH_3_COO)- | alpha-ketoisovaleric acid | 3.03 | 7.54E-02 | Short-chain keto acids and derivatives |
| (M-H)- | Citrate | 0.20 | 5.94E-03 | Tricarboxylic acids and derivatives |
| (M+H)+ | Ile-Leu | 0.28 | 3.35E-02 |  |
| (M+H)+ | Pro-Arg | 2.16 | 1.07E-02 |  |
| (M+H)+ | Norharmane | 2.58 | 5.36E-05 |  |

^a^Fold changes are mean values of six bioreplicates

Table S9 Summary of significantly changed metabolites identified in *P. putida* KT2440

| Adduct | Description | Fold change^a^ | Padj | Subclass |
| --- | --- | --- | --- | --- |
| M+ | Trigonelline | 0.141311 | 5.23E-06 |  |
| (M-H)- | sn-Glycerol 3-phosphoethanolamine | 0.195289 | 4.66E-05 |  |
| (M-H)- | DL-3-Phenyllactic acid | 0.219529 | 3.4E-06 |  |
| (M-H)- | DL-lactate | 0.391779 | 0.004393 | Alpha hydroxy acids and derivatives |
| (M+H)+ | L-Histidine | 0.174883 | 0.025524 | Amino acids, peptides, and analogues |
| (M+H)+ | 3-Aminobutanoic acid | 0.215155 | 0.000106 | Amino acids, peptides, and analogues |
| (M+H)+ | L-Aspartate | 0.277101 | 4.76E-05 | Amino acids, peptides, and analogues |
| (M+H)+ | NG,NG-dimethyl-L-arginine | 0.349004 | 0.000365 | Amino acids, peptides, and analogues |
| (M+H)+ | L-Proline | 0.42371 | 0.04051 | Amino acids, peptides, and analogues |
| (2M+Na)+ | L-Lysine | 0.424188 | 0.000381 | Amino acids, peptides, and analogues |
| (M+H)+ | Ornithine | 0.430849 | 0.075128 | Amino acids, peptides, and analogues |
| (2M+Na)+ | N6-Methyl-L-lysine | 0.451757 | 0.000659 | Amino acids, peptides, and analogues |
| (M+H)+ | L-Citrulline | 2.503675 | 0.005998 | Amino acids, peptides, and analogues |
| (M+H)+ | N-Acetylglutamine | 3.843567 | 0.000147 | Amino acids, peptides, and analogues |
| (M+CH_3_CN+H)+ | L-Pyroglutamic acid | 5.466657 | 4.87E-05 | Amino acids, peptides, and analogues |
| (M-H)- | D-Aspartic acid | 0.219061 | 4.58E-05 | Amino acids, peptides, and analogues |
| (M-H)- | Acetyl-DL-Leucine | 0.227512 | 0.000145 | Amino acids, peptides, and analogues |
| (M-H)- | L-Phenylalanine | 2.523554 | 2.38E-05 | Amino acids, peptides, and analogues |
| (M+H)+ | Anthranilic acid | 0.497522 | 1.03E-05 | Benzoic acids and derivatives |
| (M-H)- | Formylanthranilic acid | 0.386916 | 0.000523 | Benzoic acids and derivatives |
| (M+H-2H_2_O)+ | N-Acetyl-D-glucosamine | 0.110032 | 9.4E-05 | Carbohydrates and carbohydrate conjugates |
| (M-H_2_O-H)- | Ribitol | 0.185752 | 0.000142 | Carbohydrates and carbohydrate conjugates |
| M- | Glycerol | 0.217874 | 2.35E-05 | Carbohydrates and carbohydrate conjugates |
| (M-H)- | DL-2-Phosphoglycerate | 0.217874 | 0.002476 | Carbohydrates and carbohydrate conjugates |
| (M-H)- | N-Acetylglucosamine 1-phosphate | 0.236462 | 4.29E-05 | Carbohydrates and carbohydrate conjugates |
| (M-H_2_O-H)- | L-Sorbose | 0.312829 | 0.003171 | Carbohydrates and carbohydrate conjugates |
| (M-H)- | D-Mannose | 0.317993 | 0.004175 | Carbohydrates and carbohydrate conjugates |
| (M-H)- | Succinate | 0.294446 | 3.61E-06 | Dicarboxylic acids and derivatives |
| (M-H)- | 2-Methyl-3-hydroxybutyric acid | 0.172024 | 0.000472 | Fatty acids and conjugates |
| (M-H)- | Hydroxyisocaproic acid | 0.200534 | 0.000228 | Fatty acids and conjugates |
| (M-H)- | 3-Hydroxyisovaleric acid | 0.30886 | 0.000561 | Fatty acids and conjugates |
| (M+H)+ | 3-Methylbutanoyl-CoA | 0.113316 | 0.000461 | Fatty acyl thioesters |
| (M-H)- | Indole | 0.295537 | 0.002961 | Indoles |
| (M+H-H_2_O)+ | 5-Hydroxyindoleacetate | 0.319008 | 0.000546 | Indolyl carboxylic acids and derivatives |
| (M-H)- | L-Tryptophan | 0.330423 | 1.73E-06 | Indolyl carboxylic acids and derivatives |
| (M-H)- | 3-Hydroxycapric acid | 0.188727 | 0.00038 | Medium-chain hydroxy acids and derivatives |
| (M+H-H_2_O)+ | Tyramine | 3.181958 | 0.000789 | Phenethylamines |
| (M+H)+ | Guanosine 5'-diphosphate | 7.64639 | 7.15E-05 | Purine ribonucleotides |
| (M-H)- | Uracil | 2.0884 | 0.011028 | Pyrimidines and pyrimidine derivatives |
| (M+H)+ | L-Carnitine | 0.369096 | 8.15E-05 | Quaternary ammonium salts |
| (M-H)- | Citrate | 0.396235 | 0.027289 | Tricarboxylic acids and derivatives |
| (M+H)+ | Ile-Ala | 0.017425 | 0.002729 |  |
| (M+H)+ | Methoprene | 0.195148 | 0.00011 |  |
| (M+H)+ | Val-Ala | 0.195585 | 2.84E-05 |  |
| (M+H)+ | L-NG-Monomethylarginine | 0.298748 | 7.78E-05 |  |
| (M+H)+ | sn-glycero-3-phosphoethanolamine | 0.357044 | 6.36E-05 |  |
| (M+H-H_2_O)+ | Pro-Ser | 0.417811 | 0.007233 |  |
| (M+H)+ | Arg-Asp | 0.426517 | 0.000504 |  |
| (M+H-H_2_O)+ | Pro-Val | 0.497235 | 0.03311 |  |
| (M-H)- | alpha-N-Acetyl-L-glutamine | 3.113876 | 0.000794 |  |

^a^Fold changes are mean values of six bioreplicates

Table S10 Summary of significantly changed metabolites identified in *P. fluorescens* ATCC13525

| Adduct | Description | Fold change^a^ | Padj | Subclass |
| --- | --- | --- | --- | --- |
| (M+H)+ | Adenine | 0.41 | 1.42E-02 |  |
| (M+H)+ | all cis-(6,9,12)-Linolenic acid | 9.69 | 2.41E-05 |  |
| (M+H)+ | Triethanolamine | 2.05 | 4.61E-03 | Alkanolamines |
| (M+CH_3_COO+2H)+ | L-Pyroglutamic acid | 0.45 | 1.04E-03 | Amino acids, peptides, and analogues |
| (M+H-H_2_O)+ | N-Acetyl-L-glutamate | 0.48 | 5.39E-04 | Amino acids, peptides, and analogues |
| (M-H)- | N-Acetyl-L-glutamate | 0.44 | 6.19E-04 | Amino acids, peptides, and analogues |
| (M+NH_4_)+ | Cellobiose | 2.14 | 1.33E-02 | Carbohydrates and carbohydrate conjugates |
| (M-H)- | DL-2-Phosphoglycerate | 0.38 | 3.28E-03 | Carbohydrates and carbohydrate conjugates |
| (M+CH_3_COO)- | D-Mannose | 0.46 | 7.67E-02 | Carbohydrates and carbohydrate conjugates |
| (M-H)- | 3-Hydroxydodecanoic acid | 3.59 | 3.25E-02 | Medium-chain hydroxy acids |
| (M-H)- | Phosphoenolpyruvate | 0.39 | 7.26E-03 | Phosphate esters |
| (M+H)+ | Cytosine | 2.17 | 5.98E-02 | Pyrimidines and pyrimidine derivatives |

^a^Fold changes are mean values of six bioreplicates

Fig. S1 The concentration of pyoverdine presenced in the *P. aeruginosa* cultures.

Pyoverdine concentration was quantified as described by Filloux and Ramos (2014). Bacterial cells were pelleted by centrifugation at 10,000×g for 10 min at 4℃, and the clear supernatants were diluted in 50 mmol L^-1^ pyridine-acetic buffer (pH 5.0). The concentration was calculated using the absorbance recorded at 380 nm and the molar extinction coefficient of pyoverdine (ε = 16,500 mol^-1^.cm^-1^).
